# Cancer stem cells are underestimated by standard experimental methods in clear cell renal cell carcinoma

**DOI:** 10.1038/srep25220

**Published:** 2016-04-28

**Authors:** Craig Gedye, Danylo Sirskyj, Nazleen C. Lobo, Jalna Meens, Elzbieta Hyatt, Michael Robinette, Neil Fleshner, Robert J Hamilton, Girish Kulkarni, Alexandre Zlotta, Andrew Evans, Antonio Finelli, Michael A. S. Jewett, Laurie E. Ailles

**Affiliations:** 1 School of Biomedical Sciences and Pharmacy, University of Newcastle, University Drive, Callaghan, NSW 2308, Australia; 2 Princess Margaret Cancer Centre, University Health Network, Toronto, ON, M5G 1L7, Canada; 3 Hospital for Sick Children, Program in Genetics and Genome Biology, Toronto, ON, M5G 0A4, Canada; 4 Dept. of Medical Biophysics, University of Toronto, Toronto, ON, M5G 1L7, Canada

## Abstract

Rare cancer stem cells (CSC) are proposed to be responsible for tumour propagation and re-initiation and are functionally defined by identifying tumour-initiating cells (TICs) using the xenotransplantation limiting dilution assay (LDA). While TICs in clear cell renal cell carcinoma (ccRCC) appeared rare in NOD/SCID/IL2Rγ^−/−^ (NSG) mice, xenografts formed more efficiently from small tumour fragments, indicating the LDA underestimated ccRCC TIC frequency. Mechanistic interrogation of the LDA identified multiple steps that influence ccRCC TIC quantitation. For example, tissue disaggregation destroys most ccRCC cells, common assays significantly overestimate tumour cell viability, and microenvironmental supplementation with human extracellular factors or pharmacological inhibition of anoikis increase clonogenicity and tumourigenicity of ccRCC cell lines and primary tumour cells. Identification of these previously uncharacterized concerns that cumulatively lead to substantial underestimation of TICs in ccRCC provides a framework for development of more accurate TIC assays in the future, both for this disease and for other cancers.

Cancers are epigenetically and genetically heterogeneous^1^. There are several proposed mechanisms for epigenetic heterogeneity including phenotypic plasticity, epithelial-mesenchymal transition and the cancer stem cell (CSC) hypothesis^2^. The CSC hypothesis posits hierarchies within cancers wherein rare isolatable cancer cells can exclusively self-renew, differentiate and extensively proliferate to repopulate primary tumours or establish metastatic lesions. The therapeutic implication of this is that rare CSC may have unique properties not shared by the bulk of the tumour cells^3^ and may thus represent under-appreciated therapeutic targets.

The CSC hypothesis is functionally tested by the xenotransplantation limiting dilution assay (LDA). A range of tumour cell doses is injected into cohorts of mice, and Poisson statistics are used to calculate the frequency of cells capable of initiating xenografts. Modifications of assay conditions have however led to dramatic differences in tumour-initiating cell (TIC) frequencies. In melanoma TIC frequencies went from as few as 1 in 10^6^ cells^4^ to 1 in 4 cells upon assay optimization^5^. Conversely TICs appear to be rare in other tumour types even under these optimized conditions^6^. This highlights the central controversy surrounding the CSC hypothesis; if TICs are not rare (*i.e.* if the majority of cancer cells can reinitiate tumours), then most cancer cells will share tumour-perpetuating biological programs, and the CSC hypothesis will have little clinical relevance, whereas if TICs are rare, it remains important to identify, isolate and characterize these cells.

Others and we have previously discussed methodological concerns at a variety of experimental stages when interrogating the CSC hypothesis, but noted that these have been incompletely explored^7^,^8^. CSCs have been reported in clear cell renal cell carcinoma (ccRCC) using cultured cells^9^, but we sought to investigate ccRCC CSC using primary patient tumours. TICs initially seemed rare in ccRCC samples using the gold-standard xenotransplantation method, but high engraftment with small, unprocessed tumour fragments contradicted this result and prompted us to interrogate the accuracy of the LDA. We found multiple sources of mechanistic error that lead to substantial underestimation of the clonogenic and tumourigenic potential of ccRCC cancer cells. The magnitude of these inaccuracies has significant implications for the identification and enumeration of TICs in ccRCC and suggests a need for rigorous re-evaluation of methods used to quantify TICs in other solid tumours as well.

## Results

### Orthotopic limiting dilution assays indicate TICs are rare in ccRCC samples

**Figure 1:**
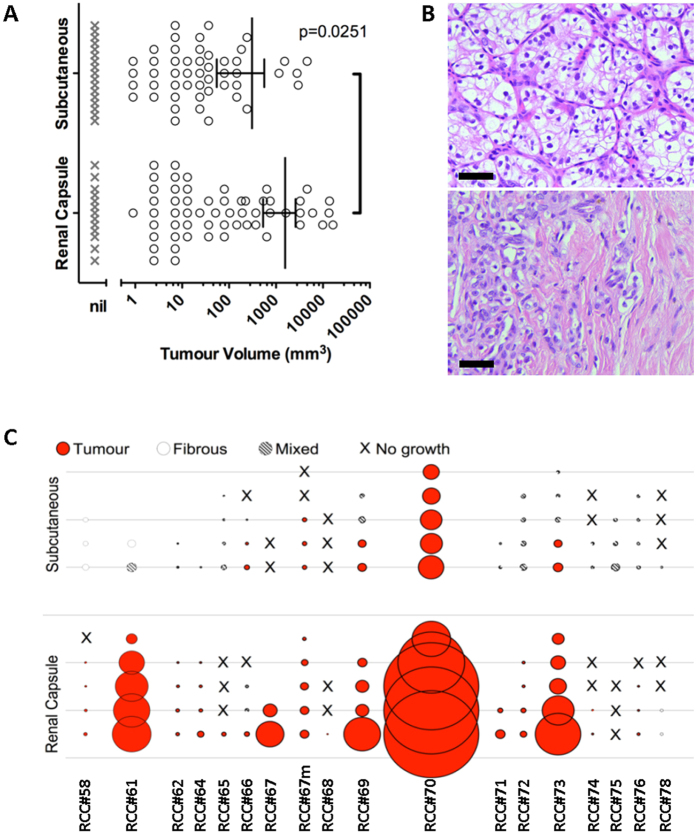
ccRCC xenografts in the renal capsule are larger and recapitulate the histology of ccRCC better than xenografts in the subcutaneous space of NSG mice. (**A**) ccRCC appears to engraft with similar frequency in the subcutaneous (17/18) and renal capsule (28/30) niches, however the mean xenograft volume was larger (p = 0.0251) in the subrenal capsule space (circles represent macroscopic xenografts; crosses represent mice in which xenografts did not form; data are represented as mean ± SEM). (**B**) Haematoxylin and eosin staining of matched xenografts grown in subrenal capsule *vs*. subcutaneous space (RCC#62, scale bar = 50 μm). (**C**) Matched patient xenografts forming in the renal subcapsular space (bottom) formed larger masses, comprised of typical ccRCC tumour cells compared to subcutaneous xenografts (top), which were more likely to be composed of fibrous stroma. Area of circle represents calculated volume of tumour; texture represents character of xenograft tissue.

Patient samples employed in this study are listed in Supplementary Table 1. To optimize xenograft assays of ccRCC, we implanted small tumour fragments (1 mm^3^) from surgically resected ccRCC samples in either the renal subcapsular space or subcutaneously in NSG mice. Mice were assessed for engraftment after 6 months or earlier if mice were morbid/had palpable tumours. Xenografts formed with a similar frequency of >90% at both sites, but were larger in the subcapsular *vs.* the subcutaneous space (Fig. 1A), and subcapsular xenografts recapitulated patients’ clear-cell histology (Fig. 1B), whereas subcutaneous implantation resulted in generally smaller masses that often partially or wholly consisted of fibrous connective tissue (Fig. 1B,C and Supplementary Figure 1). The renal capsule niche was therefore employed for all subsequent experiments.

**Figure 2:**
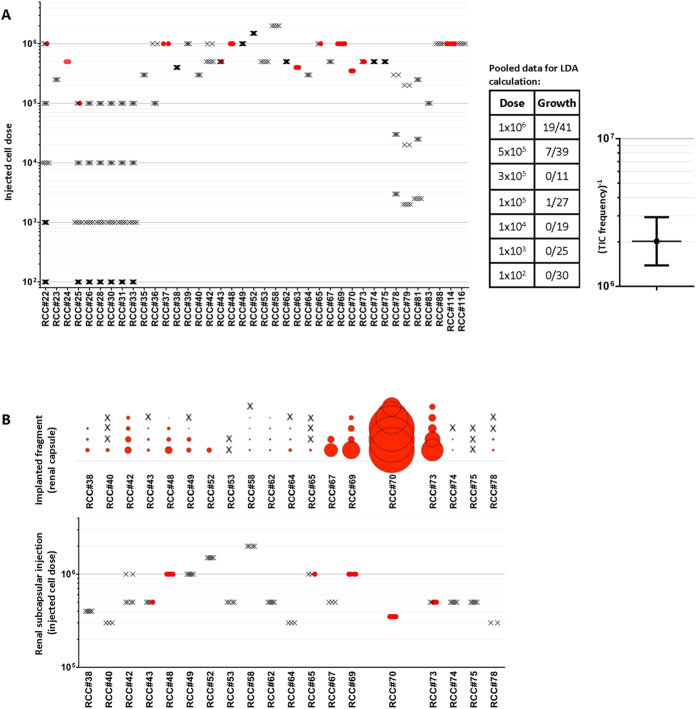
Tumour fragments of ccRCC engraft more efficiently than single cell suspensions in NSG mice. (**A**) Left panel: Mice were injected under the renal capsule with *ex vivo* ccRCC single cell suspensions with cell doses ranging from 100 to 2 million. The minimum dose for successful tumour initiation ranged from 1 × 10^5^ to 1 million cells. Each marker represents an individual injected mouse; red circles represent successful xenografts, crosses xenografts that failed to form. Middle panel: Table of pooled results showing engraftment rates (# of mice engrafted/# of mice injected) at each dose. Only doses for which >10 mice per dose were injected were included. Right panel: A Poisson statistics calculation of the pooled data indicates an average TIC frequency of 1 in 2 million (confidence interval 1 in 1.4 million to 1 in 2.9 million, n = 33 patients). Horizontal line indicates the estimated TIC frequency and the bars indicate 95% confidence intervals. These data suggest that TICs in ccRCC are very rare. (**B**) 1 mm^3^ tumour fragments of ccRCC engraft more efficiently than high dose single cell suspensions in NSG mice. Comparison of matched samples injected as tumour fragments and single cell suspensions. Area of circle in top graph represents calculated volume of tumour. Tumours that were unable to engraft as single cell suspensions were often able to engraft as fragments.

We generated single cell suspensions from primary patient tumours to quantitatively assay ccRCC TIC frequency at doses ranging from 10^2^ to 2 × 10^6^ cells. Xenografts formed from 12/30 patients’ cancers (40%), but in only three cases at cell doses less than 5 × 10^5^ viable cells (Fig. 2A), suggesting that TICs were rare in ccRCC. We next performed Poisson statistics analysis of these results using the Extreme Limiting Dilution Analysis (ELDA) algorithm (http://bioinf.wehi.edu.au/software/elda/)^10^ to generate TIC frequency estimates with 95% confidence intervals (CI). Due to the small numbers of animals engrafting per individual patient, we performed this analysis on pooled data, using only doses for which at least 10 mice were injected per dose. The average TIC frequency in ccRCC samples was calculated to be 1 in 2 million cells (95% CI, 1 in 1.4 million to 1 in 2.9 million; Fig. 2A). The low efficiency of engraftment obtained with single cell suspensions was in striking contrast to the 93% engraftment rate of small fragments (28 of 30 samples engrafted). In matched cases almost all samples engrafted from fragments (17/19) but only 6/19 samples formed xenografts from a high dose injection (3.5 × 10^5^ to 2 × 10^6^ cells; Fig. 2B).

### Tumour disaggregation causes substantial tumour cell destruction

To determine if the discrepancy between tumour fragments and cell suspensions was due to differences in the total number of cells implanted, we estimated the total number of cells per unit tissue volume based on Hoechst nuclear staining of tissue sections from twenty patient samples. We calculated a mean of 1.74 × 10^5^ total cells per mm^3^ (range 5.5 × 10^4^ to 4.5 × 10^5^; Supplementary Figure 2A), in close agreement to previous estimates^11^. We then focused specifically on the number of cancer cells within fragments *vs*. cell suspensions. Tumour cell frequencies in solid tissues were determined by immunofluorescence staining with a pan-Cytokeratin (CK) antibody to allow quantitation of the CK-positive cells; the average tumour cellularity was 65.7% (Supplementary Figure 2A,B). By contrast, flow cytometry analysis for CD45^+^ immune cells and CD45-/CK + epithelial cells in single cell suspensions indicated that the average frequency of cancer cells was 33.4% (range 2.9% to 80%; Supplementary Figure 2C,D). The downward shift in cancer cellularity observed in single cell suspensions *vs*. tumour tissue suggested that ccRCC tumour cells were being selectively lost during processing, which is supported by the observation that tumour digestion often generated a layer of lipid on top of the supernatant during washing steps that is likely due to lysis of the lipid-containing “clear” ccRCC cells (Supplementary Figure 2E). Taking these numbers into account, we calculated that the mean number of tumour cells implanted in tumour fragments was 1.13 × 10^5^ (range 2.5 × 10^4^ to 2.25 × 10^5^) and the mean number of tumour cells injected in what was thought to be a 5 × 10^5^ cell dose was 1.7 × 10^5^ (range 1.5 × 10^4^ to 4 × 10^5^; Table 1). We were also able to estimate our total tumour cell recovery by comparing the number of cells obtained per cm^3^ of tissue against the calculated cell number per cm^3^ in *ex vivo* patient samples, finding that as few as 1.25% of ccRCC tumour cells survive the disaggregation process. In summary, we illustrate the extraordinary impact of generating cell suspensions on tumour cell viability in ccRCC, and demonstrate that a 5 × 10^5^ cell dose contained similar numbers of cancer cells as a 1 mm^3^ tumour fragment.

### Humanization of the mouse microenvironment increases ccRCC cell line tumourigenicity and promotes clear cell histology

Even though high dose (3.5 × 10^5^ to 2 × 10^6^) cell suspension injections contained similar tumour cell doses to tissue fragments, the significantly higher engraftment efficiency with fragments suggested that other variables were impacting the poor tumourigenicity of cell suspensions. We hypothesized that tissue digestion with proteolytic enzymes followed by multiple washing steps was removing essential human extracellular matrix (ECM) components and growth factors that are essential for tumour cell engraftment, and that the murine ECM components in Matrigel^TM^ were not optimally restoring the human TIC niche. The impact of functional incompatibility between murine and human extracellular factors and cell surface receptors is almost entirely unknown, but several growth factors and cytokines have been shown to be non-functional between mice and humans^12^,^13^. We therefore tested whether provision of factors derived from human sources, such as serum and cancer-associated fibroblasts (CAFs), could increase *in vitro* clonogenicity and *in vivo* engraftment efficiency of ccRCC single cell suspensions.

**Figure 3:**
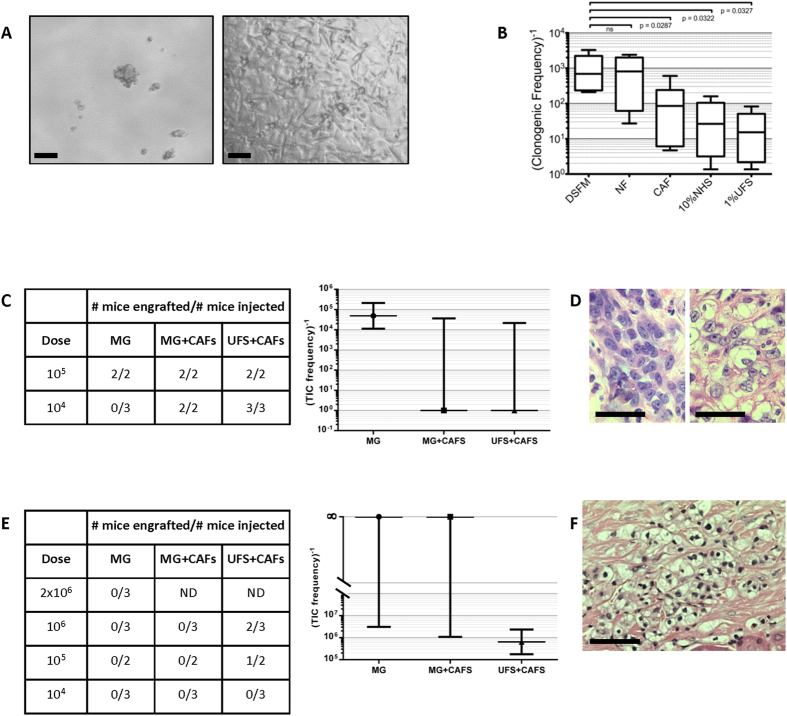
Humanization alters assay outcomes both *in vitro* and *in vivo*. (**A**) Phase contrast microscopy of 786-O cells in DSFM (left) and DSFM supplemented with 10% NHS (right; scale bars = 50 μm). (**B**) Clonogenic frequency measured by LDA of 786-O and early passage in-house ccRCC cell lines is increased when serum-free media is conditioned on cancer-associated fibroblasts (CAFs) or with addition of 10% NHS or 1% UFS. DSFM media conditioned on human normal fibroblasts (NF), human umbilical vein endothelial cells (HUVECs) or mouse embryonic fibroblasts (MEFs) did not alter clonogenicity (B. and data not shown). Cell lines 786-O, RCC#22, RCC#65, RCC#126, RCC#128, and RCC#130 were used. Box plots represent the median and range of the values obtained. (**C**) 786-O cells initiated tumors at a lower dose when co-injected with CAFs or CAFs + UFS. Left: Table of engraftment rates at each dose for each group. Right: Poisson statistics calculation of the results shown in the table. Horizontal lines indicate the estimated TIC frequencies and the bars indicate 95% confidence intervals. The estimated TIC frequency shifted from 1 in 50,000 to 1 in 1 with the addition of CAFs or CAFs + UFS. (**D**) Co-injection of CAFs + UFS leads to more clinically relevant clear cell differentiation in the 786-O cell line (right), compared to 786-O cells alone (left; scale bars = 50 μm). **(E**) Injection of the RCC#22 cell line in Matrigel alone never gave rise to xenografts even at a dose of 2 million cells, but it was tumorigenic with co-injection of CAFs and UFS. Left: Table of engraftment rates at each dose for each group. Right: Poisson statistics calculation of the results shown in the table. Horizontal lines indicate the estimated TIC frequencies and the bars indicate 95% confidence intervals. The estimated TIC frequency shifted from 1 in infinity to to 1 in 600,000 with the addition of CAFs and UFS. (**F**) RCC#22 cell line xenografts also recapitulate clear cell histology of ccRCC (scale bar = 100 μm).

Established ccRCC cell lines were tested for their ability to grow in conditions traditionally used to assay stem-like cells *in vitro*, *i.e.* defined serum free media (DSFM) containing B27 supplement, EGF and FGF^14^. Commercial and our own patient-derived early passage ccRCC cell lines had a mean clonogenic frequency in DSFM of 1 in 690 (range 1 in 210 to 1 in 3300 cells; Fig. 3). The mean clonogenic frequency was increased by up to 45-fold with the addition of various human extracellular factors such as 10% normal human serum, 1% ultra-filtrated normal human serum (UFS) or “pre-conditioning” of the DSFM on human oral squamous cell carcinoma-derived CAFs^15^ (Fig. 3B). DSFM conditioned on normal oral fibroblasts, human umbilical vein endothelial cells or early passage mouse embryonic fibroblasts had no effect on clonogenicity (data not shown).

Human extracellular components also increased ccRCC cell line tumourigenicity in NSG mice. The 786-O cell line was tumourigenic in Matrigel alone, but the minimum tumourigenic dose was reduced by ≥10-fold with the addition of CAFs, or a combination of CAFs and UFS (Fig. 3C). Indeed, the use of these additives led to growth of this cell line in every mouse injected, leading to a TIC estimate of 1 in 1 with a one-sided confidence interval (Fig. 3C), in significant contrast to the result obtained with MG alone (TIC estimate of 1 in 50,000, CI 1 in 11,000 to 1 in 212,000). Interestingly, the phenotype of 786-O xenografts took on typical clear cell histology in the presence of human UFS and human CAFs, as compared to a bland undifferentiated phenotype of xenografts in the presence of Matrigel alone (Fig. 3D). Furthermore, the early passage RCC#22 cell line was tumourigenic only in the presence of UFS and CAFs (Fig. 3E,F). RCC#22 even at high doses (2 × 10^6^) with Matrigel alone was never tumourigenic (yielding a TIC frequency estimate of 1 in infinity and a one-sided confidence interval), but xenografts formed at doses of 1 × 10^5^ to 1 × 10^6^ in the presence of UFS + CAFs (yielding a TIC frequency estimate of 1 in 640,000, CI 1 in 175,000 to 1 in 2.3 million; Fig. 3E). This cell line recapitulates the *VHL* mutation and somatic copy number alteration profile of the patient’s primary tumour (data not shown). These data show that “humanization” of the microenvironment can quantitatively and qualitatively influence the tumourigenic potential of human ccRCC cell lines.

### Humanization does not influence *ex vivo* ccRCC cell suspension tumourigenicity

*Ex vivo* patient ccRCC cell suspensions were co-injected in either Matrigel alone or in Matrigel plus UFS + CAFs. In these experiments patient samples were also depleted of CD45^+^ cells (to more accurately quantitate TIC frequency). The maximum tumour cell dose injected was 1 × 10^5^ cells in most cases due to limited cell numbers, which is comparable to the calculated cell doses in the 1.5 × 10^4^–4 × 10^5^ range described above for undepleted single cell suspensions. Engraftment was again only observed in cases where very high cell doses were injected (Supplementary Figure 3A). Use of human placental ECM in combination with Matrigel also failed to influence the tumourigenic frequency (Supplementary Figure 3B).

We hypothesized that CD45^+^ cell depletion might be causing loss of tumourigenic potential since immune cells are pro-tumourigenic in many contexts. Depleted CD45^+^ cells were mixed back with CD45^neg^ (tumour) cells, at cell doses ranging up to 4 × 10^5^ ccRCC cells. Engraftment was again patient-dependent, only doses greater than 3 × 10^5^ were tumour-initiating and CD45^+^ cells had no differential influence (Supplementary Figure 3C). Thus, broad “humanization” of the murine microenvironment had an impact on clonogenicity and tumourigenicity in ccRCC cell lines, but could not normalize tumourigenicity of *ex vivo* ccRCC suspensions to the levels seen with implanted tumour fragments.

Mechanical stiffness of the injection matrix improves xenotransplantation in some cancers^16^, but induces differentiation and growth arrest in other contexts^17^. We did not prospectively test this *in vivo*, but in retrospective *in vitro* experiments, cell lines able to proliferate in Matrigel concentrations from 50% upward are those that are robustly tumourigenic (786-O, #243 cell lines) whilst poorly tumourigenic and more slowly proliferative cell lines (RCC#22, #207 and #371) were clonogenic only in 25–33% Matrigel (Supplementary Figure 4). The concentration of Matrigel used for almost all tumourigenicity experiments in our work and published CSC studies is 50%.

### Dye exclusion methods grossly overestimate ccRCC cell suspension viability

**Figure 4:**
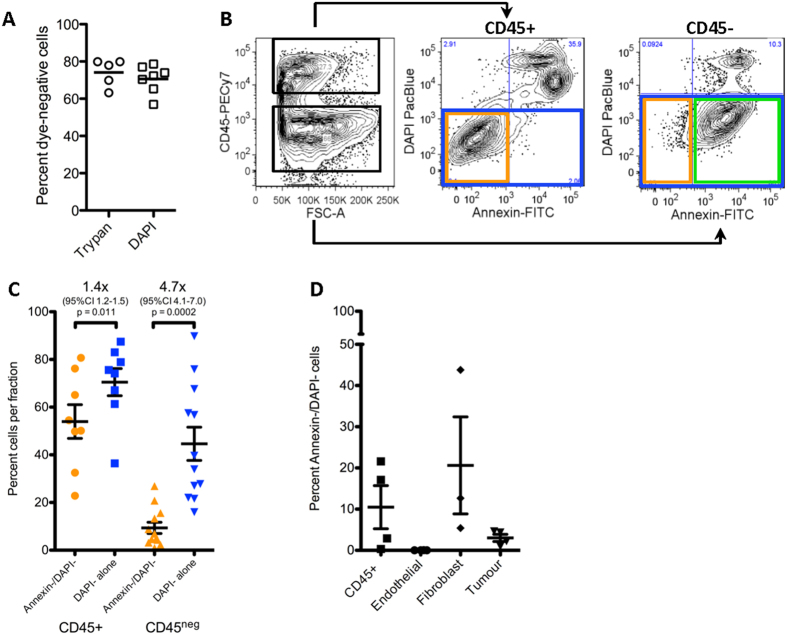
Trypan blue or DAPI exclusion overestimates tumour cell viability which is heterogeneous in tumoural subpopulations. (**A**) Trypan blue exclusion and DAPI exclusion give equivalent estimates of cell viability in matched and unmatched fresh primary patient samples. (**B**) In freshly processed *ex vivo* ccRCC patient samples, cell viability is overestimated by DAPI exclusion alone, as a high proportion of DAPI-negative cells are Annexin-V positive (green box). Truly viable Annexin-V^−^/DAPI^−^ cells are in the left lower quadrant (orange box). (**C**) Comparison of viability assessment in CD45^+^ and CD45^neg^ fractions using DAPI alone (blue) or DAPI and Annexin-V (orange). DAPI alone significantly overestimates viability, by ~4.7X (p = 0.0002) in the CD45^neg^ compartment. Each point represents an individual patient. (**D**) A deeper examination of viability in different tumour cell sub-compartments reveals that while CD45^+^ cells and fibroblasts have reasonable viability, endothelial cells were uniformly apoptotic or dead. Tumour cell viability remained very low in these experiments. Data in C and D are represented as mean ± SEM. See Supplementary Figure 6 for gating strategy.

When we placed primary single cell suspensions in culture, we noted that thawed cryopreserved cells appeared to have acceptable post-thaw viability by trypan blue exclusion, but almost completely failed to grow *in vitro*. Optimization of our thawing protocol did not appear to alter trypan blue exclusion but functional recovery was greatly improved, equivalent to freshly processed cells (Supplementary Figure 5). These observations suggested that trypan blue does not accurately quantify cell viability, which could contribute to inaccuracies in the readouts of quantitative clonogenic and *in vivo* TIC assays. To more accurately assess cell viability, primary ccRCC cells were stained with Annexin-V-FITC and DAPI immediately after processing or after thawing using our optimized protocol (see Methods). While DAPI staining tended to show similar proportions of viable and dead cells as trypan blue exclusion (Fig. 4A), Annexin-V co-staining revealed that the majority of CD45-negative DAPI-negative cells (*i.e.* “viable cancer cells”) were Annexin-V-positive, and thus in early apoptosis (Fig. 4B, lower right quadrant). Only a fraction of DAPI^neg^ CD45^neg^ cells were Annexin-V negative (1–20%; Fig. 4C). For the samples analysed, viability was overestimated by 4.1- to 7-fold (mean 4.7-fold) using DAPI staining alone. Conversely CD45^+^ cells within each sample showed almost equivalent viability as measured by DAPI or DAPI and Annexin-V (only a 1.4X difference). Co-staining of ccRCC samples with additional cell surface markers revealed that CD45^neg^CD31^+^ CD34^+^ endothelial cells were almost completely non-viable, whereas TE7^+^ fibroblasts^18^ had similar viability to CD45^+^ cells (Fig. 4D).

Ammonium chloride red cell lysis buffer can be toxic to nucleated blood cells^19^ so we hypothesized that this final step in tumour processing may trigger apoptosis in ccRCC cells. However, there was no difference in Annexin-V/DAPI staining or *in vitro* clonogenicity when this step was omitted (data not shown).

### Anoikis contributes to apoptosis in ccRCC cell suspensions

**Figure 5:**
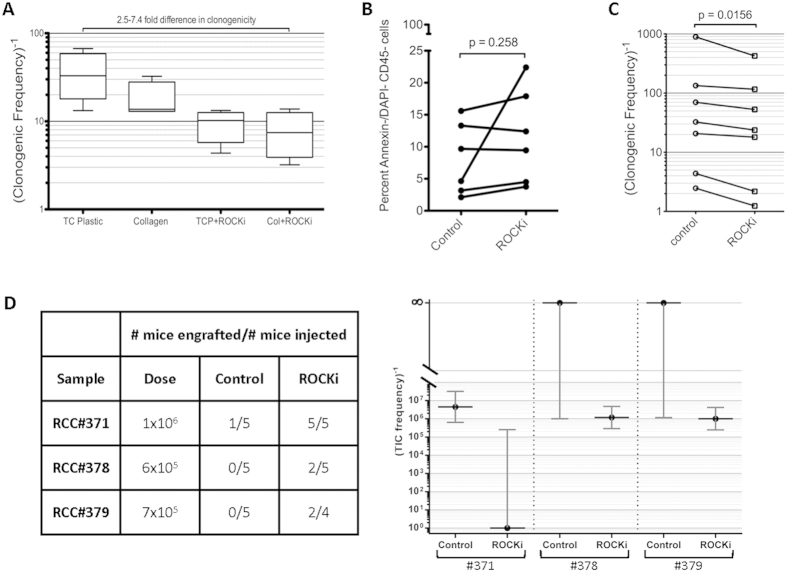
Inhibition of anoikis increases viability and clonogenicity in ccRCC cell lines and primary samples. (**A**) Treatment of ccRCC cell lines (#22, #207, #323, #371) with the ROCK-1 inhibitor Y-27632 increases clonogenic frequency by 2.5–7.4 fold. The addition of a collagen substrate *vs*. tissue culture plastic (TCP) alone could only partially rescue this loss of clonogenic potential (confidence intervals estimated by ELDA). Box plots represent the median and range of the values obtained. (**B**) Primary tumour processing in the presence of Y-27632 showed a trend to increased viability in *ex vivo* primary human ccRCC samples as detected by DAPI/Annexin-V staining. (**C**) Inhibition of anoikis *ex vivo* increases clonogenicity. Clonogenic assays on primary tumour single cell suspensions digested with Y-27632 showed higher clonogenic frequency (~1.6 fold) than control cell suspensions (p = 0.0156). (**D**) Inhibition of anoikis *ex vivo* increases tumourigenicity. *Ex vivo* patient ccRCC samples were processed and injected into the sub-capsular space of NSG mice, in the presence or absence of Y-27632 at all points during the experimental protocol. While a high dose of injected cells in one patient sample (#371) was able to form a xenograft, tumourigenicity was more robust in cells processed with the ROCK-inhibitor, and in other patients’ cases, only cells processed with the ROCK1-inhibitor were tumourigenic. Horizontal lines indicate the estimated TIC frequencies and the bars indicate 95% confidence intervals.

We hypothesized that anoikis (apoptosis induced by detachment of anchorage-dependent cells from their matrix) contributed to apoptosis during tumour dissociation. We added an inhibitor of anoikis, the Rho-associated protein kinase-1 (ROCK1) inhibitor Y-27632, to early passage ccRCC cell lines before typical trypsin passaging and noted a significant increase in clonogenic frequency, suggesting that ccRCC cells are sensitive to anoikis *in vitro* (Fig. 5A). Provision of a collagen substrate to aid in cell adhesion only partially rescued clonogenicity, suggesting that anoikis is initiated upon cell detachment. Dissociation of *ex vivo* primary ccRCC samples showed a trend (p = 0.258) to an increased proportion of Annexin-V^neg^/DAPI^neg^/CD45^neg^ cells in the presence of 10 μM Y-27632 (Fig. 5B). ROCK1 inhibition significantly increased the clonogenic frequency (p = 0.0156; Fig. 5C), and tumourigenic potential of primary *ex vivo* ccRCC samples (Fig. 5D). Importantly the addition of Y-27632 also increased *ex vivo* clonogenicity in non-clear cell kidney cancers, including papillary, chromophobe and urothelial carcinomas (Supplementary Figure 7), suggesting that dissociation-induced anoikis is not restricted to the clear cell subtype.

### Some tumour cell apoptosis occurs before tissue disaggregation

Cleaved-caspase-3 and TUNEL staining was performed on selected ccRCC that had been implanted as fragments and injected as suspensions to determine the proportion of dead or apoptotic cells present prior to sample processing. These showed considerable variability between specimens, ranging from 7–75% (mean 30%) TUNEL positive cells and 1–90% (mean 15%) cleaved-caspase-3 positive cells. Samples that failed to engraft from injected suspensions but that still formed xenografts from fragments had high levels of apoptosis in the pre-processing sample compared to tumours that could form xenografts from injected suspensions (Supplementary Figure 8). Thus at least some of the cell death seen in suspensions occurs in patients’ samples before processing.

If we take all of our measurements of cell viability and apply them back to the original xenograft LDA assays, we find that the input cell numbers are significantly lower than they initially appeared; a cell dose initially thought to contain 5 × 10^5^ cells in fact contained approximately 3.5 × 10^4^ viable cancer cells (See Table 1). This represents an average 14.3-fold overestimate of input cell numbers, and thus 14.3-fold underestimate of TIC frequency. However, due to the wide ranges in cellularities and viabilities seen from one sample to the next, in some cases significantly higher (up to 170X) underestimations of TIC frequencies are likely. Furthermore, we find that tumour fragments contain approximately the same number of viable cancer cells as a 5 × 10^5^ dose of cell suspension. Thus it remains true that cell suspensions are less efficient at tumour initiation than tumour fragments, even when viable cancer cell numbers are accounted for, indicating that additional factors are negatively influencing TIC readouts from single cell suspensions.

## Discussion

Methodological refinements in murine xenotransplantation LDA assays such as orthotopic or empirically optimized injection sites^20^, co-injection with Matrigel, use of more immunocompromised mice, and long observation times^5^ have led to significantly altered readouts in CSC assays. We report further, multiple, previously unexplored experimental errors that impede accurate quantification of TICs in the solid tumour ccRCC.

Initial experiments showed that tumour fragments implanted orthotopically in the subrenal capsule yielded larger xenografts that better recapitulated the clear cell histology of ccRCC *vs*. subcutaneous implants. Subsequent LDA injection experiments were therefore performed orthotopically, augmented by previously reported TIC assay refinements; Matrigel co-injection, NSG mice, and 6-month incubation to ensure accurate accounting of engraftment^5^.

The high efficiency of xenograft initiation from tumour fragments is consistent with other reports of human primary ccRCC xenografts^21^,^22^,^23^. To perform a quantitative Poisson analysis of single cell suspension engraftment data, we pooled together data from a large number of samples. While this provided an average TIC frequency across samples rather than individual TIC frequencies among patients, this approach provides adequate numbers for a more accurate TIC frequency calculation. This calculation showed that the average TIC frequency in ccRCC single cell suspensions is 1 in 2 million. The poor tumorigenicity of single cell suspensions was discordant with the high tumourigenicity of small fragments and suggested inaccuracies in the assay. This led us to more accurately enumerate the number of actual cancer cells in fragments and cell suspensions. Cytometric analysis showed a disproportionately large fraction of stromal cells in suspensions compared to that predicted from histology and pan-CK immunofluorescence, most likely due to selective destruction of cancer cells during processing. This suggested that injected high cell doses contained similar numbers of cancer cells to tumour fragments and thus TIC frequency estimates were on average 3-fold and up to 100-fold higher than previously appreciated. Furthermore, even when taking cancer cellularity into account, the higher engraftment efficiency of fragments (93%) *vs*. high dose single cell suspensions (40%) indicates that the LDA assay still underestimates TIC frequency in ccRCC samples.

We hypothesized that ECM lost during dissociation was not restored by Matrigel or the murine niche. Previous observations indicating a lack of cross-reactivity between murine ligands and human receptors^12^,^13^ suggested that human tumour extracellular signalling may be dysfunctional in the murine microenvironment. This hypothesis is supported by many reports that provision of human growth factors and/or stromal cells improves xenotransplantation of human cancers^24^,^25^,^26^,^27^,^28^,^29^,^30^. We pragmatically attempted to “humanize” the mouse microenvironment with CAFs, ultra-filtrated human serum and ECM, as these substantially altered ccRCC cell line clonogenicity *in vitro*. CAF and/or UFS co-injection increased or enabled tumourigenicity of ccRCC lines and also gave tumours that recapitulated the clear cell histology of primary ccRCC, but failed to alter tumourigenicity from *ex vivo* primary ccRCC single cell suspension injections. One explanation for this discrepancy is that provision of human extracellular factors and/or CAFs upon injection is too late to counteract the apoptosis that was initiated during dissociation (discussed below). Cultured cell lines take little time to mobilize to suspensions, and are likely better adapted to resist anoikis and might therefore be more responsive to additional human factors.

Annexin-V staining revealed that the majority of DAPI-negative tumour cells were apoptotic, and that most viable cells were CD45^+^ leukocytes, which has been previously unappreciated in the CSC literature using typical viability assessment tools (trypan blue or DAPI exclusion). The contrast between CD45^+^ and CD45^neg^ Annexin-V staining within each sample acts as an internal control, mitigating against the possibility that Annexin-V staining overestimates apoptosis in digested fresh tissue samples.

Tumour digestion with hyaluronidase and collagenase liberates cells from ECM contact and may therefore contribute to anoikis, thus accounting for at least some of the apoptotic cancer cells present in single cell suspensions. ROCK1 inhibition increases the survival of cancer cells^31^, embryonic stem cells^32^ and murine prostate stem/progenitor cells^33^ and we similarly found that ROCK1 inhibition throughout dissociation and processing increased *in vitro* clonogenicity and *in vivo* tumourigenicity of dissociated patient ccRCC samples. TUNEL and cleaved Caspase-3 staining demonstrated variable numbers of apoptotic cells prior to tumour dissociation, at levels somewhat higher than previously reported^34^, and while pre-dissociation viability appeared to be associated with subsequent tumourigenic potential, the number of apoptotic cells in fragments was not associated with delays in time to processing, and thus were likely an intrinsic property of individual patient samples.

While it is believed that in some cancers only a proportion of cells that are clonogenic *in vitro* have the potential to initiate tumours *in vivo* (*i.e.* most clonogenic cells are “progenitor” or “transit-amplifying” cells), in others this is not the case. For example, in murine malignant peripheral nerve sheath tumours^35^ it was shown that assay conditions significantly influenced readouts of both tumourigenic and clonogenic assays, and once these assays were optimized, both clonogenic and tumourigenic cells were found to be common. During the course of optimizing culture conditions for ccRCC we observed high clonogenic frequencies from primary ccRCC samples (range 1 in 2 to 1 in 400; Fig. 5C). Based on our calculations in Table 1, these clonogenic frequencies are approximately 200-fold higher than tumourigenic cell frequencies. Indeed, even in cell lines, where many of the issues relating to viability and anoikis are reduced, there is a great difference between tumourigenic and clonogenic cell frequencies (Supplementary Figure 9). However, we cannot currently determine whether this reflects two functionally distinct populations within these cell lines, or the fact that the TIC assay remains suboptimal. Functional experiments to assay the tumour-initiating ability of individual clonogenic cells are required to determine if indeed this is the case. However, our findings and previous reports underscore the complexity of interpretation of these assays, and highlight the importance of studying clonogenic and tumourigenic cells in parallel.

We are aware of additional variables that we have not addressed in this work, such as residual mouse immunity. We did not explore different mouse strains, but studies in several other tumour types^6^,^20^,^36^ have compared TIC assays in NSG *vs*. NOD/SCID mice. While these did not show significant differences in these small studies, when these data are taken as a whole there is a statistically significant (mean ~4-fold) increase in tumourigenicity in NSG mice compared to the previous standard NOD/SCID mouse model (Supplementary Figure 10), suggesting that the immune status of the recipient mouse can significantly influence TIC readouts. While the NSG mouse is currently one of the most immunocompromised models for xenograft assays, the remaining innate immunity in these mice may still negatively influence TIC readouts. Furthermore, it has been shown that human hematopoietic stem cells engraft more efficiently in female as compared to male mice^37^, and our study used only male mice.

An additional consideration is the poor cell recovery of only 1.25% of cancer cells upon tumour dissociation; it remains unknown whether there is a selection bias in the surviving cells that would introduce even further inaccuracies into the TIC assay, or in which direction resulting readouts may be skewed. The degree of mechanical stiffness in the injection matrix is also noted to be influential^38^, and in limited experiments we were intrigued to note that only cell lines able to proliferate in Matrigel concentrations from 50% upward are robustly tumourigenic whilst poorly tumourigenic cell lines were clonogenic only in 33% Matrigel and below. The concentration of Matrigel used for almost all tumourigenicity experiments in this work and others is 50%. These preliminary observations have not been explored *in vivo* but merit deeper investigation in all TIC assays, as although Matrigel improves xenotransplantation in some cancers^16^, it also induces differentiation and growth arrest in other contexts^17^.

**Figure 6:**
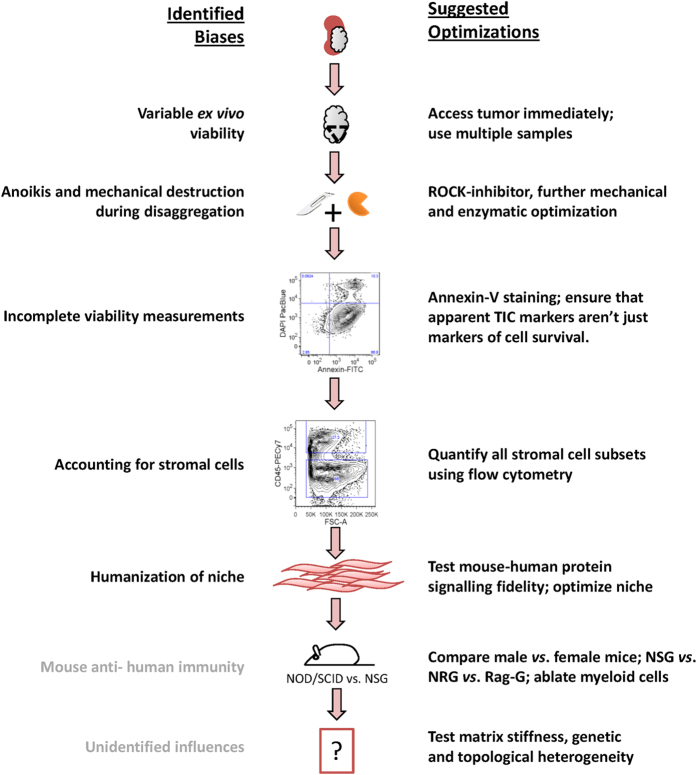
Summary of identified biases and suggested optimizations. We show that better assessment of cell viability, accurate accounting for stromal cells, and humanization of the tumour microenvironment can substantially alter tumour TIC readouts in ccRCC (black text). Additional variables not assessed in our study likely contribute even further to TIC underestimates (grey text). Some of these concerns are addressable (for example, by appropriately accounting for stromal populations and accurately measuring viable cells) and others can be partially remediated (for example by adding ROCK inhibitor to circumvent apoptosis during cell dissociation). However, several consequences of tissue dissociation, such as loss of cell-cell contact, clear cell friability and selective destruction, and removal from the *in vivo* human microenvironment, remain unavoidable and difficult to resolve, and their influences on TIC readouts remain unknown.

In conclusion, our results indicate that typical tumour processing and cell counting protocols used to perform quantitative xenotransplantation assays, as well as incompatibilities in the xenograft microenvironment, can lead to substantial underestimation of TIC frequency in ccRCC. Wide variations in stromal and tumour cell numbers and viabilities, combined with inter-patient molecular heterogeneity, suggest that TIC frequencies are likely heterogeneous in ccRCC. We were able to influence the TIC frequency by at least 10-fold by co-injecting human extracellular factors which would bring the number of viable cancer cells required to initiate tumours down to an average of 3500, but in some cases as few as 300 cells may be sufficient (Table 1). It is therefore conceivable that TICs may not be rare in ccRCC, and the question of whether ccRCC follows the hierarchical model or not remains an open question. Cancer cell friability might be specific to ccRCC, but it is likely that most if not all the concerns we have identified are relevant to TIC xenotransplantation assays in other tumours, *e.g.* the effect of anoikis inhibition in non-clear cell kidney cancers (Supplementary Figure 7). Some of these issues are addressable (*e.g.* accounting for stromal populations and accurate viability assessments) and others can be partially remediated (*e.g*. ROCK1 inhibition of anoikis), but additional optimizations are required to develop truly accurate TIC assays. We provide a novel framework to improve current methods for the interrogation of the CSC hypothesis that should be applied not only to ccRCC, but to all solid tumour CSC studies (Fig. 6).

## Methods

### Tumour collection and processing

Primary ccRCC samples were obtained from the University Health Network (UHN) Program in Biospecimen Sciences and from the Cooperative Health Tissue Network (CHTN) from consenting patients in accordance with the policies of the UHN Research Ethics Board. All samples were dissected immediately after surgery to exclude necrotic and non-viable areas of tumour, and histologically verified by a pathologist (Supplementary Table 1). Tumours were procured within 2–24 hours of excision. In some cases 1 mm^3^ fragments were collected for immediate xenograft implantation. Remaining samples were dissociated into single cell suspensions. For dissociation, tumours were minced with sterile scalpels and digested with 1X collagenase/hyaluronidase (Stem Cell Technologies) and DNase I (125 U/mL, Invitrogen) in defined serum-free media (see below) plus antibiotics at 37 °C for a maximum of two hours with frequent gentle trituration as previously described^39^. Red blood cells were lysed by applying 1 mL of ammonium chloride lysis buffer (Gibco) to the cell pellet for 5 minutes on ice, followed by an immediate wash in DMEM + 10% foetal bovine serum (FBS). Following red cell lysis, cells were filtered through a 70-micron sterile nylon mesh and viable cells defined by trypan blue exclusion were enumerated at 10× magnification by haemocytometer. Cells were cryopreserved by slow freezing in DMEM containing 10% FBS and antibiotics plus 10% dimethylsulfoxide in a CoolCell® container (Biocision). An optimized thawing protocol was developed in which cells were rapidly thawed, then diluted carefully and slowly to 10× volume by iterative drop-wise addition of 10% volumes of DMEM + 10% FBS. In some experiments, tumours were evenly divided in two, with one fragment processed with the Rho kinase inhibitor Y-27632 (10 μM^31^,^32^,^33^,^40^; Tocris Bioscience) present at all steps including washes, lysis, cryopreservation, thawing, dilution and cell culture.

### Cell line establishment and maintenance

The 786-O cell line was a gift from Dr. Michael Ohh, University of Toronto. RCC#22, RCC#65, RCC#126, RCC#128, RCC#130, RCC#207, RCC#323 and RCC#371 cell lines were established in our laboratory from primary patient tumour samples^41^. In-house cell lines were verified by targeted *VHL* gene sequencing with confirmation against original patient tumour samples and were used at passage 5 or less. Established cell lines were cultured at 37 °C, 5% CO_2_ in high-glucose DMEM (Wisent, St. Bruno, Canada) supplemented with 10% FBS, 2 mM glutamine, 100 U/mL penicillin, and 100 μg/mL streptomycin, and passaged with 0.05% trypsin in 2 mM EDTA in PBS, pH 7.4 (Wisent). Routine *Mycoplasma* surveillance (MycoAlert, Lonza, Basel, Switzerland) and cell line identity verification by STR profiling (AmpFLSTR® Identifiler®, TCAG, SickKids Toronto) were performed as described previously^39^.

### Flow cytometry

Cells were stained and analysed using fluorescence-minus-one (FMO) controls and single-colour stained compensation beads (BD Biosciences) on a BD LSR II. Lineage antibodies included CD31, CD34, CD45 (BioLegend), and TE7^18^ (in-house production from ATCC hybridoma). Antibody concentrations were optimized by titration and varied from batch to batch and with different fluorochrome conjugates. Annexin-V-FITC (BioLegend) staining was modified with titration of buffer calcium concentration^42^ and use of 1 μg/ml 4′,6-diamidino-2-phenylindole (DAPI)^43^ in place of 7-aminoactinomycin. For intracellular pan-CK staining cells were first stained with a LIVE/DEAD fixable violet dead cell stain (ThermoFisher Scientific) diluted 1:1000 in PBS at room temperature for 30 minutes. Cells were then stained with lineage antibodies as described above, fixed in ice cold Cytofix (BD Biosciences) for 20 minutes on ice, then permeabilized in PBS, 0.5% BSA, 0.5% Saponin, 0.02% Sodium Azide for 20 minutes on ice prior to adding the PE-conjugated panCK antibody (AbCam) at a dilution of 1:200 and incubating on ice for 20 minutes.

### Clonogenicity

Commercial and early passage newly established ccRCC cell lines were transferred to a defined serum free medium (DSFM; 1:1 DMEM/F12, 1X B-27, 2 mM glutamine, 100 U/mL penicillin, and 100 μg/mL streptomycin (Gibco), 20 ng/mL epidermal growth factor and 20 ng/mL basic fibroblast growth factor (EMD Millipore) and cultured with multiple media changes. Clonogenic LDA assays were performed in 96 well plates at cell doses from 1 to 500 cells per well with a minimum of 5 replicate wells per cell dose and analysed by extreme limiting dilution analysis (ELDA; http://bioinf.wehi.edu.au/software/elda/). Wells were scored as positive if they contained at least 50 viable adherent cells 2 weeks after plating^44^. Examples of positive and negative wells are shown in Supplementary Figure 11. Primary ccRCC samples were treated similarly with the addition of collagen coating to cell culture plates (rat tail collagen I, Sigma-Aldrich). In some experiments cells were trypsinized and plated in the presence of 10 μM ROCK1 inhibitor Y-27632.

### Tumourigenicity

All animal studies were performed in accordance with the Canadian Council on Animal Care with the approval of the UHN Animal Care Committee. Initially bulk tumour cells (without CD45 + cell depletion) were re-suspended in 1:1 PBS/standard growth factor Matrigel (BD Biosciences) and injected under the renal capsule of male NOD/SCID/IL2Rγ^−/−^ (NSG) mice. 1 mm^3^ fragments of un-dissociated fresh ccRCC were implanted under the renal capsule or in the subcutaneous space by gentle blunt dissection. Mice were monitored by palpation for tumours for six months, and inspected at sacrifice for renal, hepatic, pulmonary and lymph node engraftment. Renal xenografts were measured and volumes were calculated according to Feldman *et al.*^45^. Limiting doses of ccRCC cell lines were co-injected with previously characterized human cancer associated fibroblasts (CAFs) and/or human ECM components derived from placenta (BD Biosciences) and/or ultra-filtrated human serum (UFS). CAFs were derived from an oral squamous cell carcinoma specimen as described in Lohse *et al.*^15^. Briefly, tumour tissue was processed as described above for ccRCC samples and cell suspensions were cultured in Iscove’s Modified Dulbecco’s Media containing 10% FBS. In these conditions squamous carcinoma cells do not survive and fibroblasts take over the cultures and can be passaged. Fibroblasts were verified by staining for pan-Cytokeratin and Vimentin, as well as by flow cytometry to exclude CD45 + immune cells and CD31 + endothelial cells. UFS was prepared by ultracentrifugation with Amicon Ultra 0.5 mL 3K centrifugal filters. Enrichment was typically around 5-fold (from ~70 mg/mL protein to ~350 mg/mL as measured by 280 nm absorbance and Bradford assay). CD45-depleted primary tumour suspensions were co-injected at two or three doses in combination with fixed doses of CAFs, or re-mixed with fixed doses of autologous CD45 + cells. In some experiments cells were injected with 25% hECM or 25% UFS in combination with 25% Matrigel. CD45 + cells were separated from tumour single cell suspensions by magnetic bead assisted cell separation according to the manufacturer’s instructions (Miltenyi Biotec) and purities confirmed as >90% by flow cytometry.

### Immunohistochemistry and immunofluorescence

Formalin-fixed paraffin embedded (FFPE) sections from xenografts were stained with haematoxylin and eosin. Cleaved-caspase-3 and TUNEL staining was performed on frozen sections by the Pathology Research Program, University Health Network, Toronto. Caspase and TUNEL positive cells were counted and scored using plugins with ImageJ^46^. Immunofluorescence was performed as described by Robertson *et al.*^47^. Briefly, five-micron FFPE sections were de-waxed, antigen retrieved with citraconic anhydride^48^, stained with pan-cytokeratin (1:200, clone C11; AbCam,), AlexaFluor594-conjugated goat-anti-mouse IgG, (1:250; Life Technologies), and Hoechst 33342 and mounted in Fluoromount (Sigma-Aldrich). Images from a Zeiss LSM700 confocal microscope were analysed by Definiens Tissue Studio. Tissue shrinkage during processing was estimated to be approximately 2-fold based on published histopathologic *vs*. radiologic co-localization data^49^,^50^,^51^. The number of cells per mm^2^ was used to calculate cells per volume of tissue (cells/mm^3^ = (cells/mm^2^ × √cells/mm^2^)/2).

### Analysis and statistics

Flow cytometry data was analysed with FlowJo (9.6.4; TreeStar), and comparisons made by paired T-test with GraphPad Prism 5.

## Additional Information

**How to cite this article**: Gedye, C. *et al.* Cancer stem cells are underestimated by standard experimental methods in clear cell renal cell carcinoma. *Sci. Rep.* **6**, 25220; doi: 10.1038/srep25220 (2016).

## Supplementary Material

Supplementary Information

## Figures and Tables

**Table 1:** Calculated cell numbers in tumour fragments *vs*. a 5 × 10^5^ dose of single cell suspension.

1 mm Fragment			Single Cell Suspension		
	Mean #	Range		Mean #	Range
Total Cell # (Nuclei)	1.74 × 10^5^	5.5 × 10^4^–4.5 × 10^5^	Total Cell # (Trypan blue)	5 × 10^5^	
Tumour Cell # (CK + cells)	1.13 × 10^5^	2.5 × 10^4^–2.25 × 10^5^	Tumour Cell # (CD45/^−^CK + cells)^a^	1.7 × 10^5^	1.5 × 10^4^ to 4 × 10^5^
Viable Tumour Cell # (TUNEL)	3.45 × 10^4^	7.5 × 10^3^ to 6.75 × 10^4^	Viable Tumour Cell # (Lin^−^Annexin-V^−^)^b^	3.5 × 10^4^	3 × 10^3^ to 8.5 × 10^4^
Viable Tumour Cell # (c-Caspase 3)	1.72 × 10^4^	3.75 × 10^3^ to 3.37 × 10^4^	Influence of Tumour Microenvironment (assume 10-fold)	3.5 × 10^3^	300 to 3.5 × 10^3^

^a^Mean %CD45-/CK + was 33.4%, range 2.9% to 80%.

^b^Calculated based on mean 4.7-fold viability overestimate (Fig. 4).
