# Supplementary material for: Cancer stem cells are underestimated by standard experimental methods in clear cell renal cell carcinoma
Source: Sci Rep. 2016 Apr 28;6:25220. doi: 10.1038/srep25220 (PMC4848484; doi:10.1038/srep25220)
Supplement: Supplementary Information [file srep25220-s1.pdf]

Supplementary Information

Cancer stem cells are underestimated by standard experimental methods in clear cell renal cell carcinoma

Craig Gedye[*,1,2], Danylo Sirskyj[2], Nazleen Lobo[2], Jalna Meens[2], Elzbieta Hyatt[3], Michael Robinette[2], Neil Fleshner[2], Robert J Hamilton[2], Girish Kulkarni[2], Alexandre Zlotta[2], Andrew Evans[2], Antonio Finelli[2], Michael AS Jewett[2], and Laurie E Ailles[*,2,4]

Includes:

Supplementary Table 1

Supplementary Figures 1 to 11

Supplementary Table 1. Patient Samples Employed in This Study

RCC#	Gender	Reported Histology	T	N	M	G	Flow cytometry	Cell line	Subcutaneous fragments	Renal capsule fragments	Renal capsule injections	Co-injections	Clonogenicity	Annexin-V	Caspase/TUNEL staining	Anoikis-ROCKi	Lysis buffer	Immunofluorescence
8	F	Clear cell	1a	X	X	2	O											
13	M	Clear cell	1b	X	X	2												O
16	F	Clear cell	2	X	X	2	O											O
20	M	Clear cell	1a	2	X	3	O											O
22	F	Clear cell	2a	X	X	3		O			O							O
23	F	Clear cell	1a	X	X	2					O							
24	M	Clear cell	2	X	X	2					O							
25	F	Clear cell	1a	X	X	2					O							
26	F	Clear cell	3b	X	X	3	O				O							O
28	F	Clear cell	3b	0	X	4					O							
30	M	Clear cell	1b	X	X	2					O							
31	M	Clear cell	1b	X	X	3					O							
35	M	Clear cell	1a	X	X	2					O							
36	M	Clear cell	3b	X	X	2					O							
37	M	Clear cell	1a	X	X	2			O		O							
38	M	Clear cell	1b	X	X	2				O	O							
40	M	Clear cell	3a	X	X	3				O	O							
42	F	Clear cell	3b	X	X	3				O	O				O			
43	F	Clear cell	3b	X	X	3	O				O				O			O
48	F	Clear cell	1b	X	X	3				O	O				O			
49	F	Clear cell	3a	1	X	3				O	O	O			O			
52	M	Clear cell	1a	X	X	3				O	O							
53	M	Clear cell	3c	X	X	3				O	O							
55	F	Clear cell	3b	X	X	3				O								
57	M	Clear cell	1a	X	X	2				O								
58	F	Clear cell	3a	X	X	2			O	O	O							
60	M	Clear cell	3a	0	X	3				O								
61	M	Clear Cell with sarcomatoid differentiation	3a	0	X	3			O	O								
62	F	Clear cell	1b	X	X	2			O	O	O							
63	M	Clear cell	3a	0	X	4					O							
64	F	Clear cell	1a	X	X	2			O	O	O							
65	F	Clear cell	3a	0	X	2		O	O	O	O	O						
66	n.s.	Clear cell	3a	0	X	3			O	O								
67	M	Clear cell	4	0	1	2			O	O	O				O			
68	F	Clear cell	2a	0	X	2			O	O								
69	F	Clear cell	3a	X	X	2			O	O	O							
70	M	Clear cell	3a	X	X	3			O	O	O				O			
71	F	Clear cell	1b	X	X	2			O	O								
72	M	Clear cell with focal rhabdoid features	3a	0	X	4			O	O								
73	F	Clear cell, with focal rhabdoid features	2a	0	1	3			O	O	O							
74	F	Clear cell	1b	X	X	2			O	O	O							
75	F	Clear cell, Gr 3/4, locally recurrent	---	X	1	4	O		O	O	O							
76	F	Clear cell	1a	X	X	3			O	O								
78	F	Clear cell	3a	X	X	3	O		O	O	O							O
79	F	ccRCC with sarcomatoid differentiation	1b	1	X	4					O							
81	M	Clear cell	1b	X	X	3					O							
83	M	Clear cell	---	X	X	2					O							
87	M	Clear cell with sarcomatoid differentiation	1b	X	1	4	O											
88	F	Clear cell	2b	X	X	2	O				O							
89	F	Clear cell	1a	X	X	2												O
94	M	Clear cell	1b	X	X	2	O											
95	F	Clear cell	3a	X	X	3	O											
99	M	Clear cell	3a	X	X	2	O											
101	F	Clear cell	1b	X	X	1	O											
110	F	Clear cell	3b	0	0	3	O											O
114	M	Clear cell	3b	0	0	3	O				O							
115	M	Clear cell	3a	1	X	3	O				O	O						
119	M	Clear cell	1b	X	X	3	O											
126	M	Clear cell	1b	X	X	3		O										
128	F	Clear cell	1b	0	X	3		O										
130	F	Clear cell	2b	X	X	3	O	O				O						O

Table S1. Patient Samples Employed in This Study (Continued)

RCC#	Gender	Reported Histology	T	N	M	G	Flow cytometry	Cell line	Subcutaneous fragments	Renal capsule fragments	Renal capsule injections	Co-injections	Clonogenicity	Annexin-V	Caspase/TUNEL staining	Anoikis-ROCKi	Lysis buffer	Immunofluorescence
132	F	Clear cell	-	-	1	2	O											
137	M	Clear cell	1b	X	X	1	O											O
138	M	Clear cell	2	X	X	2	O											O
149	M	Clear cell	1b	X	X	2	O											
152	F	Clear cell	4	0	X	2	O											
157	F	Clear cell	2a	X	X	3	O											
158	M	Clear cell	3a	0	X	3	O											
160	M	Clear cell	3a	0	X	1	O					O						
164	n.s.	Clear cell	3a	0	X	2	O											
165	F	Clear cell	1b	X	X	2	O											
169	M	Clear cell	1b	0	X	3	O					O						
171	M	Clear cell	3a	X	X	2						O						
174	F	Clear cell	3a	X	X	3	O											
175	F	Clear cell	1b	X	X	2	O											
177	M	Clear cell	1a	X	X	2	O											
178	M	Clear cell	1b	X	X	3	O											
179	F	Clear cell	1a	X	X	3	O											
187	M	Clear cell	1b	X	X	2						O						
197	F	Clear cell	1a	X	X	2	O											
200	F	Clear cell	1b	X	X	2	O											O
204	F	Clear cell	2a	X	X	2	O											
206	M	Clear cell	1a	X	X	3	O											
222	M	Clear cell	1b	0	X	2	O											
225	M	Clear cell	3b	X	X	3	O											O
226	F	Clear cell	1b	0	X	2	O											
229	F	Clear cell, metastatic to pancreas	---	X	1	---	O					O						
238	M	Clear cell	3a	X	X	2	O											O
243	F	Clear cell	4	X	1	2	O											
252	M	Clear cell	1b	X	X	3	O											O
266	F	Clear cell	3a	1	X	3	O											O
267	M	Clear cell with sarcomatoid differentiation	4	0	X	3	O											
271	M	Clear cell	1b	0	X	4	O											O
275	M	Clear cell, G3 with focal G4	2a	0	X	3/4	O											
278	F	Clear cell	2a	X	X	2	O											
279	M	Clear cell, metastatic	---	---	1	---	O											
284	M	Clear cell	2b	X	X	2	O											
285	F	Clear cell	3a	X	X	2	O											
323	M	Clear cell	1	1a	X	X		O										
371	F	Clear cell	2	1b	X	X							O	O		O		
375	M	Papillary type 1	2	1b	X	X										O		
378	M	Clear cell	2	1b	X	X								O		O		
379	F	Clear cell	1	1a	X	X							O					
380	M	Clear cell, metastatic to pancreas	---	---	1	---	O											
382	M	Chromophobe	1b	X	X	X										O		
384	M	Clear cell, metastatic to oral cavity	---	---	1	---	O											
390	F	Clear cell, metastatic to lung	1	1b	1	---	O											
404	M	Clear cell, metastatic to R adrenal gland	---	---	1	---	O											
405	F	Clear cell, metastatic	---	---	1	---	O											
410	F	Urothelial	hig	3	1	X										O		
421	M	Clear cell	ib	0	X	2							O	O			O	
422	F	Clear cell	2a	X	X	2							O	O			O	
424	M	Clear cell	1a	X	X	2							O	O			O	
425	M	Clear cell	3a	X	X	2							O	O			O	
429	M	Chromophobe	2a	0	X	---	O									O		
430	M	Clear cell	3a	X	1	3										O		
434	M	Clear cell	1a	X	X	1							O	O		O		
436	M	Clear cell, rhabdoid	1a	X	X	4							O	O		O		
437	F	Papillary	1a	0	X	---										O		
438	M	Clear cell	1b	X	X	2							O	O		O		

T: T stage; N: nodal status; M: metastasis; G: grade; X: clinical stage not known from pathology specimen; n.s.: not specified

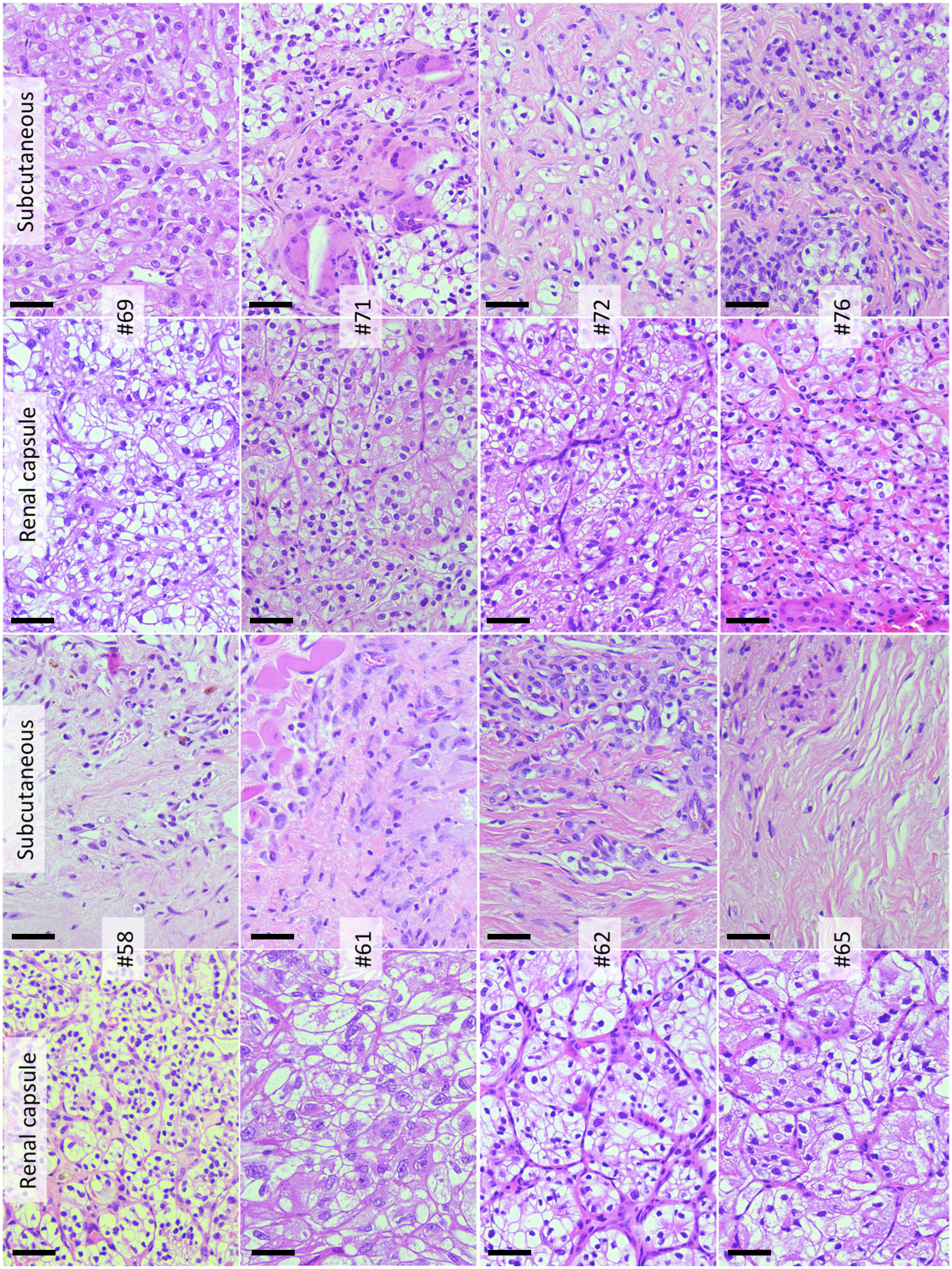

Supplementary Figure 1. Hematoxylin and eosin staining of matched xenografts formed in the renal capsule and subcutaneous space. (scale bars = 50 µm, 8 matched cases).

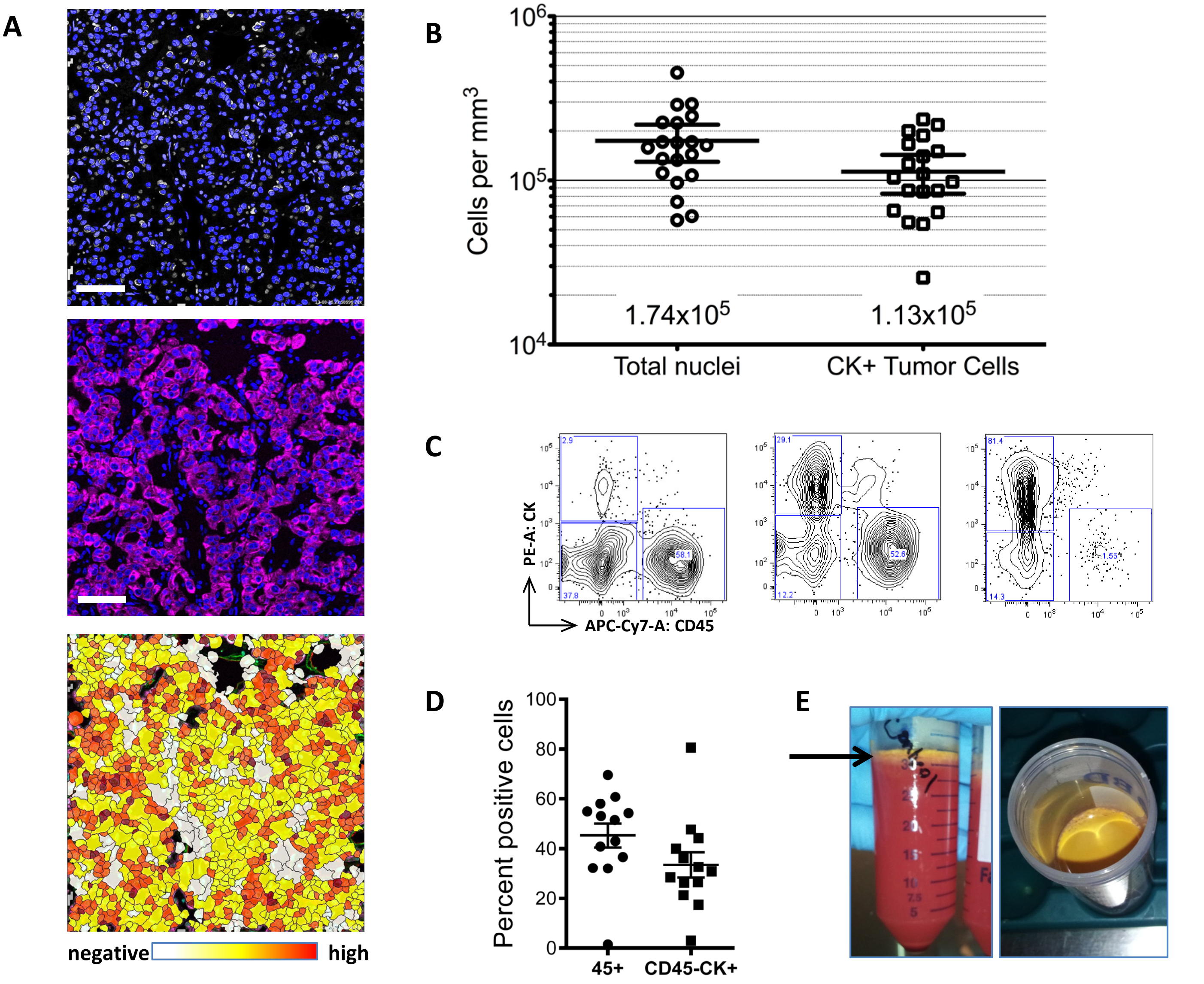

Supplementary Figure 2. Estimation of cell numbers in tumor fragments and cell suspensions.
(A) Representative ccRCC tissue section stained for nuclei with Hoescht 33342 (blue), and pan-cytokeratin (CK; pink) to estimate tumor and total cell counts (scale bar = 100 µm). Top panel, Hoechst alone; middle panel, Hoechst/CK merged image; bottom panel, example of cell counting on Definiens platform, illustrating pan-CK staining. **(B)** The number of cells per field (either total cells or CK+ cells) was used to calculate the number of cells per mm^3 of tissue. Allowing for ~2-fold shrinkage in formalin-fixed paraffin-embedded tissues[47-51] the mean number of CK+ cancer cells per mm^3 is $1.13x10^5$ (range $2.5x10^4$ to $2.25x10^5$). **(C)** To quantify the number of ccRCC cancer cells in single cell suspensions we performed intracellular flow cytometry using the same pan-CK antibody. Representative FACS plots from 3 patients, with low, medium and high proportions of pan-CK+ cells are shown. **(D)** Data summary from 13 patients analyzed as shown in (C). The mean CD45+ cell frequency in *ex vivo* single cell suspensions is 45.25% (± 4.8%; range 1.5% to 69.6%). The mean CD45-CK+ cell frequency is 33.4% (±5.1%; range 2.9% to 80%). **(E)** During processing, a lipid layer appears on the surface of the supernatant (arrow), suggesting 'clear cell' destruction. Horizontal line and error bars in (B) and (D)represent mean ± SEM.

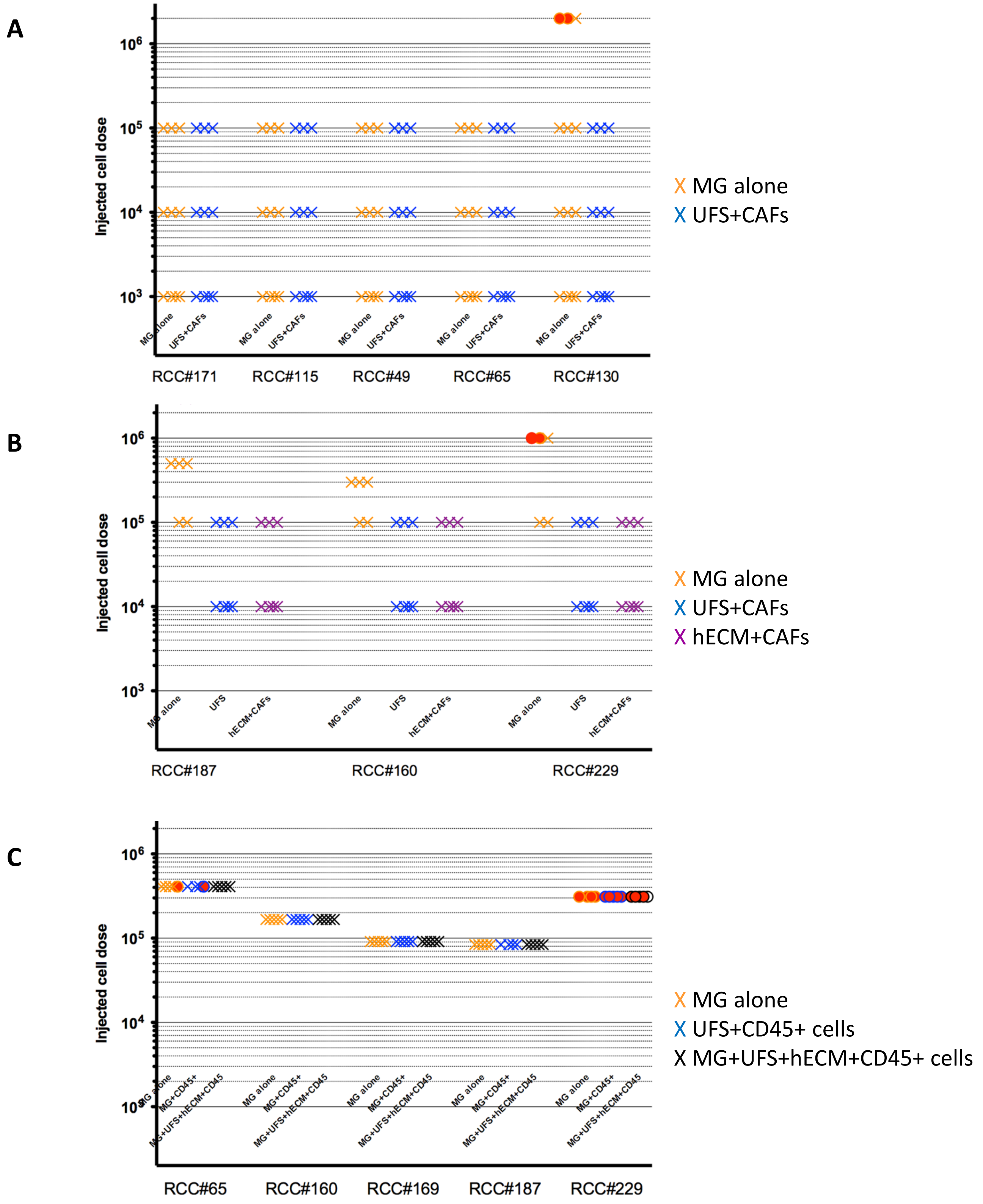

Supplementary Figure 3. "Humanization" of the mouse microenvioronment does not lower the minimum engrafting dose of primary *ex vivo* ccRCC samples. (A and B) CD45+ cell-depleted ccRCC primary tumor samples were re-supplemented with human tumor microenvironmental components such as UFS alone, CAFs and UFS (A) or CAFs and human placenta-derived extracellular matrix (B) but all failed to form xenografts except at very high doses in control conditions (Matrigel alone). **(C)** Reintroduction of separated autologous CD45+ cells (in the presence or absence of UFS or human ECM) did not influence xenograft formation.

A

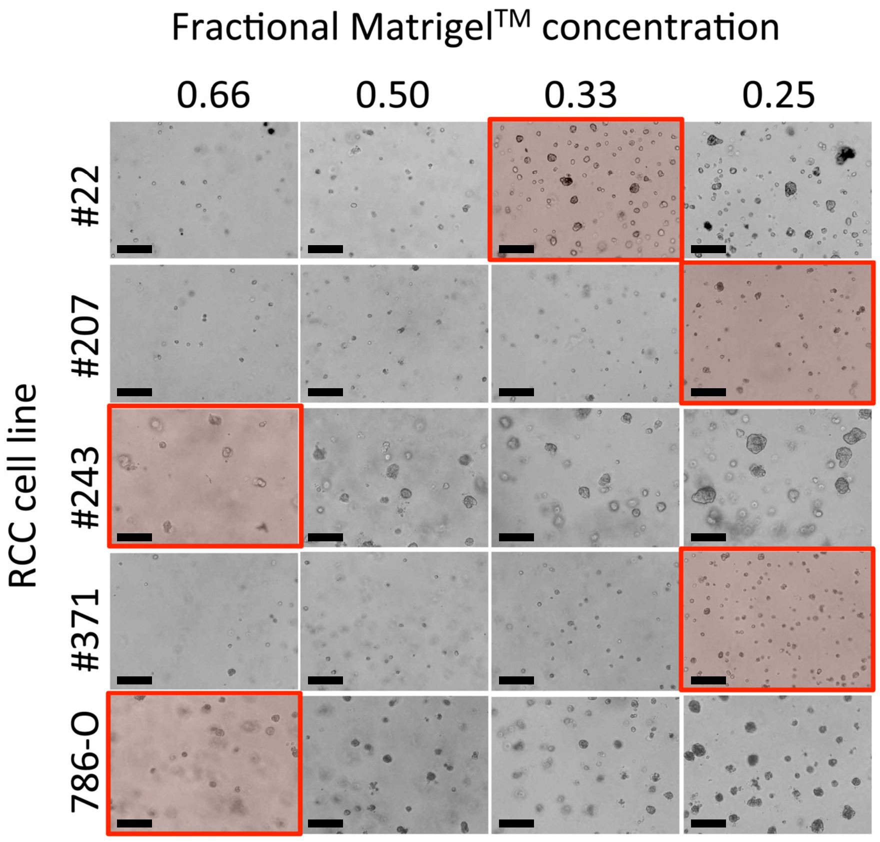

B

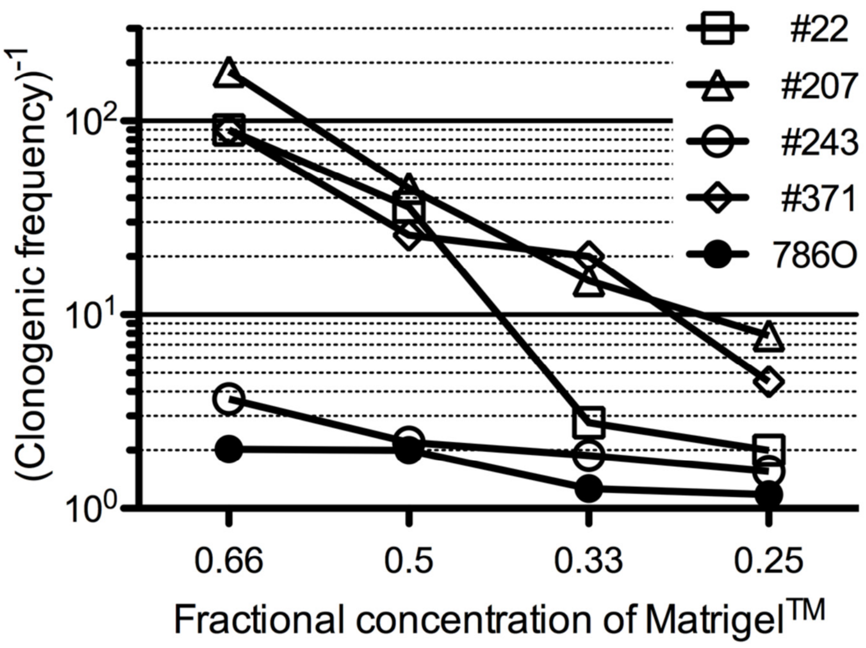

Supplementary Figure 4. Matrigel™ concentration influences ccRCC cell line clonogenicity. (A) The maximum concentration of Matrigel™ at which colonies can grow (red boxes) varies between cell lines (scale bars = 100 µm). (B) Cell lines that are known to be tumorigenic (#243, 786-O) are able to form colonies even in high fractional concentrations (0.66) of Matrigel™.

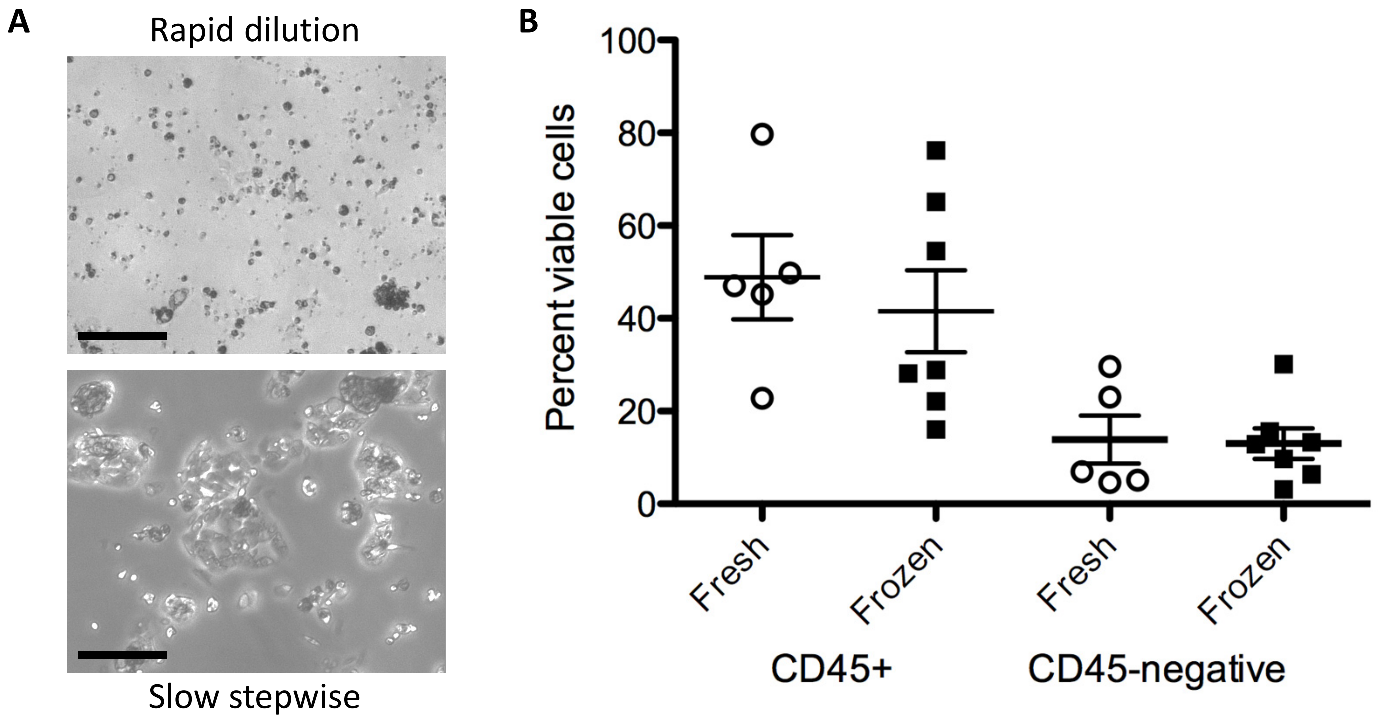

Supplementary Figure 5. Optimized thawing protocol leads to improved viability of cryopreserved *ex vivo* ccRCC samples. (A) Cell cultures following rapid thawing at 37°C and rapid dilution (top) *vs*. cells prepared by rapid thawing followed by stepwise dilution (bottom; see Methods). Slow step-wise thawing preserves cell viability (scale bar = 100 µm). **(B)** The viability of cryopreserved and optimally thawed samples is very similar to freshly dissociated tumor samples (as measured by Annexin-V-FITC negative/DAPI-negative cells). Horizontal line and error bars represent mean ± SEM.

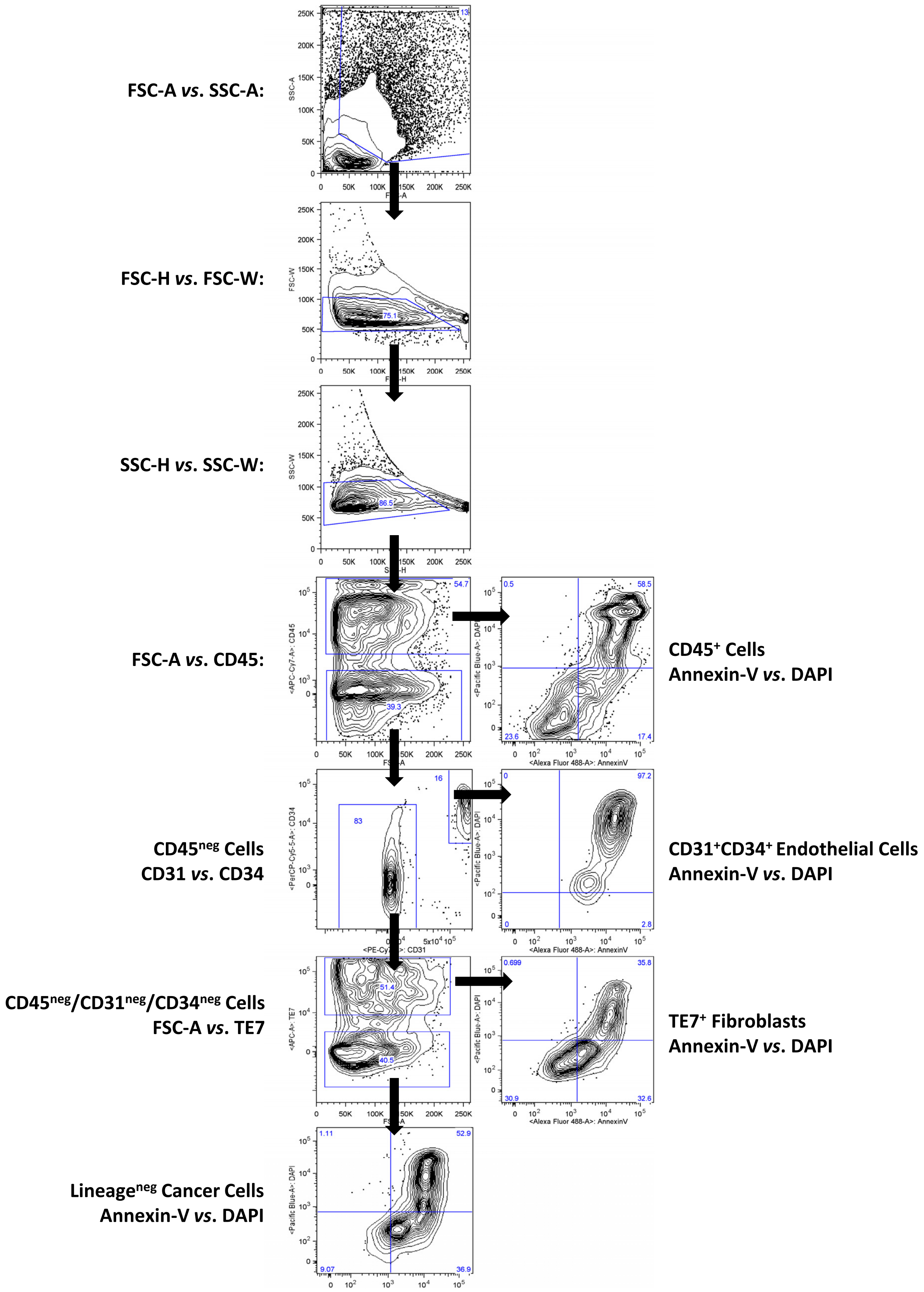

Supplementary Figure 6. Gating strategy for examining Annexin-V binding and DAPI exclusion *vs*. cell surface markers. All gates were set against fluorescence-minus-one (FMO) controls to ensure accurate gating for each population.

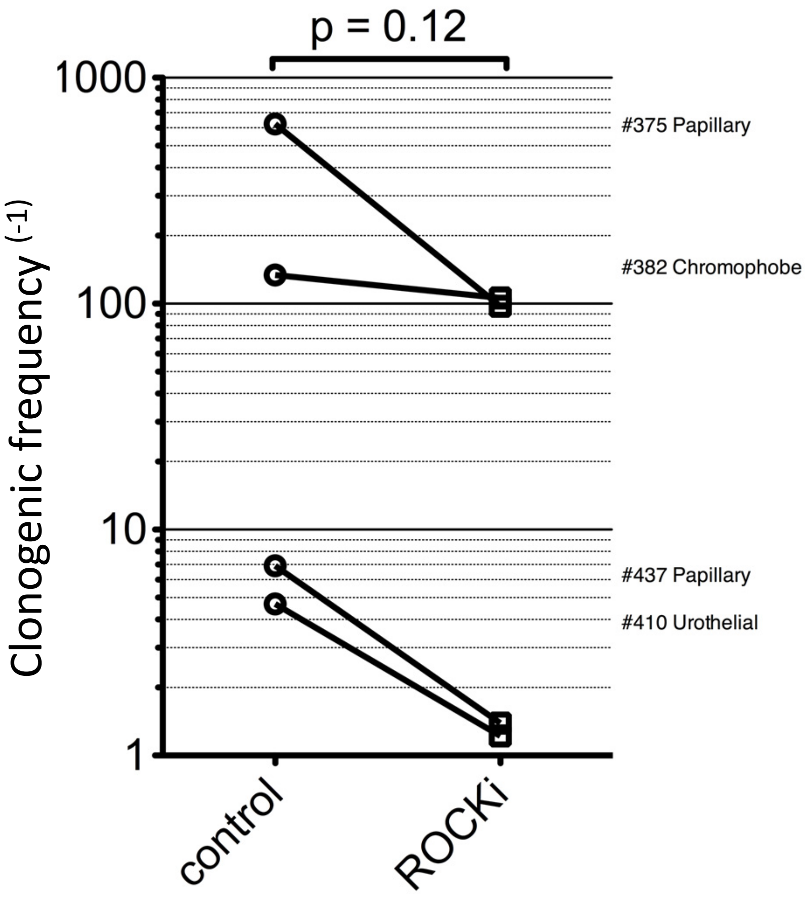

Supplementary Figure 7. Inhibition of anoikis by addition of the ROCK-1 inhibitor Y-27632 shows a trend to increased clonogenicity in non-clear cell renal carcinomas, including papillary, chromophobe and urothelial carcinomas.

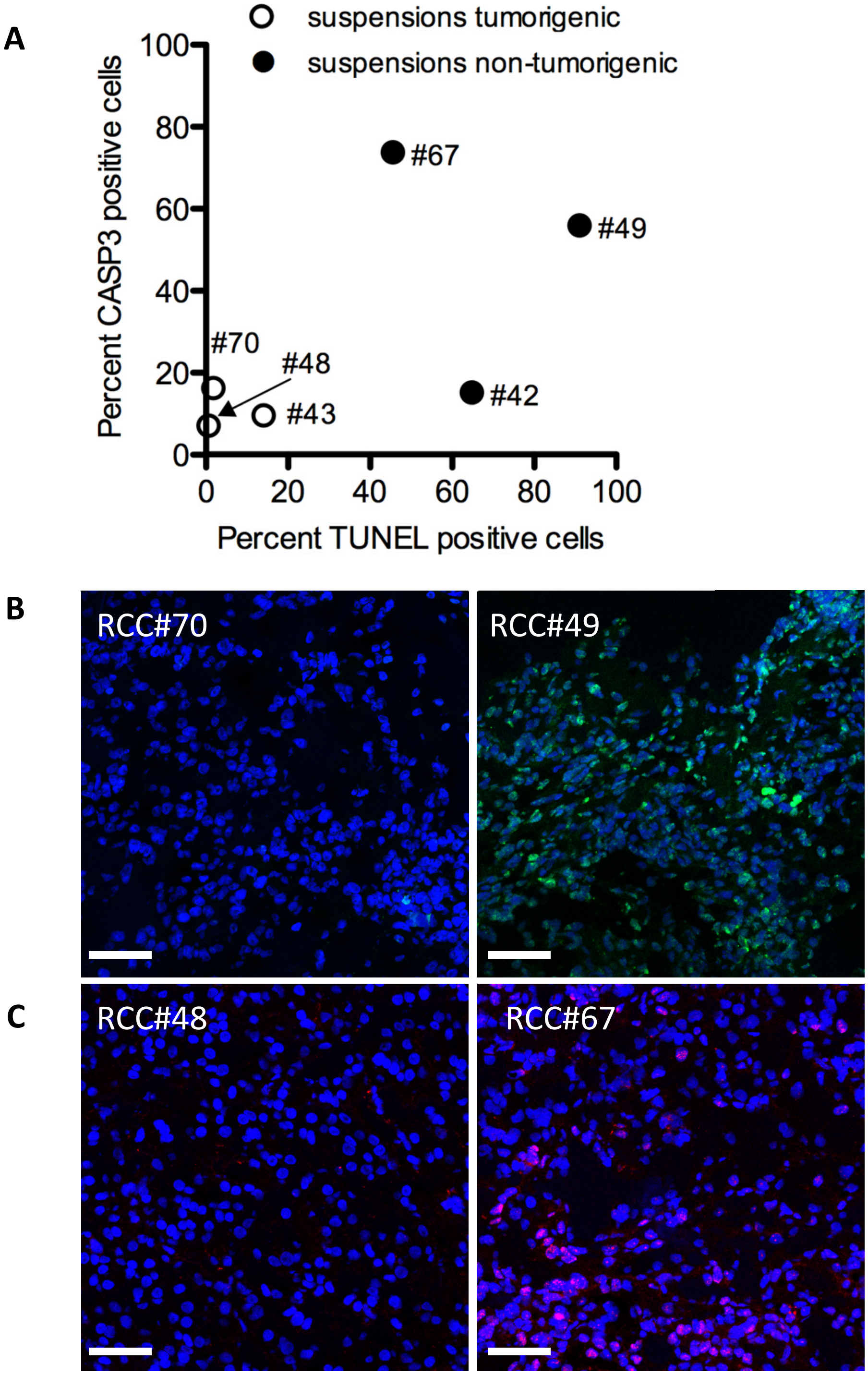

Supplementary Figure 8. Variable numbers of apoptotic cells are present in tumor tissues prior to tumor processing. (A) TUNEL and cCASP3 staining was performed on OCT embedded frozen sections of tissues adjacent to processed tumour samples to define tumour viability before tissue dissociation. A wide range of staining frequencies was observed, but interestingly samples that formed xenografts after injection of cell suspensions (#43, #48 and #70) tended to have lower TUNEL and cleaved-caspase 3 staining compared to patient samples where injections were ineffective in forming xenografts (#42, #49, #67). In all these cases xenografts formed from implanted fragments. **(B)** Examples of low (left) and high (right) TUNEL (FITC, #70, #49). **(C)** Examples of low (left) and high (right) cleaved-caspase-3 staining (Cy3, #48, #67). Scale bars = 100 µm.

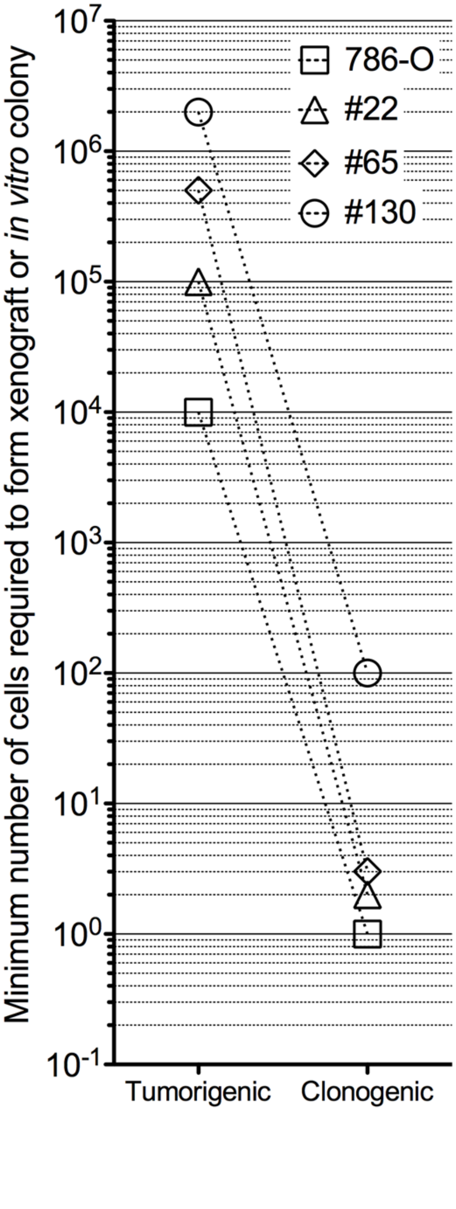

Supplementary Figure 9. Comparison of TIC and clonogenic frequencies in ccRCC cell lines.

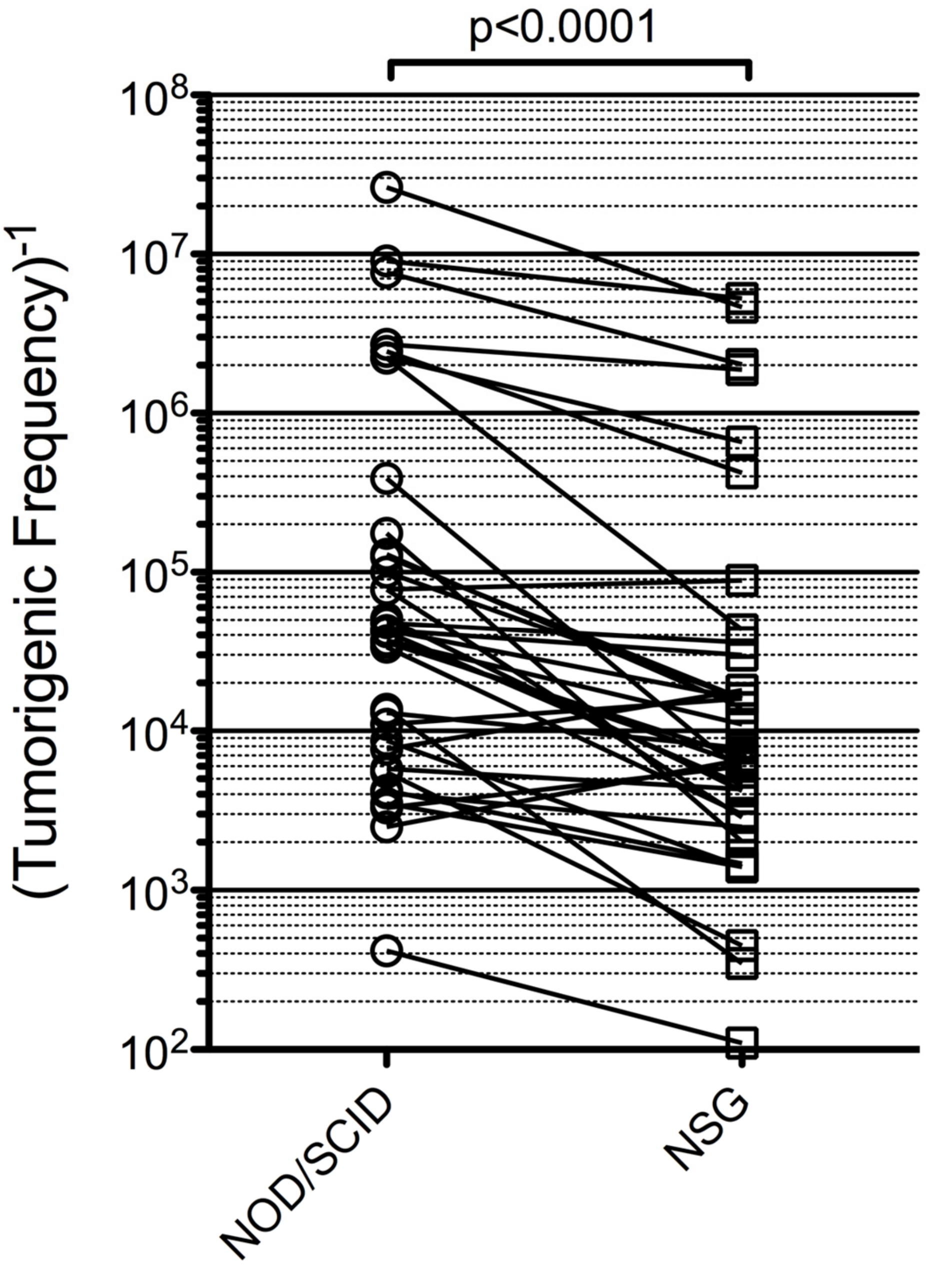

Supplementary Figure 10. The extent of mouse immunocompromisation alters TIC frequency. TIC frequency is higher if assayed in NSG versus NOD/SCID mice in ovarian carcinoma[20], acute myeloid leukemia[36], pancreatic carcinoma, non-small cell lung cancer and head and neck cancer[6]. TIC frequency was median four-fold higher if measured in NSG mice ($p<0.0001$, paired T-test).

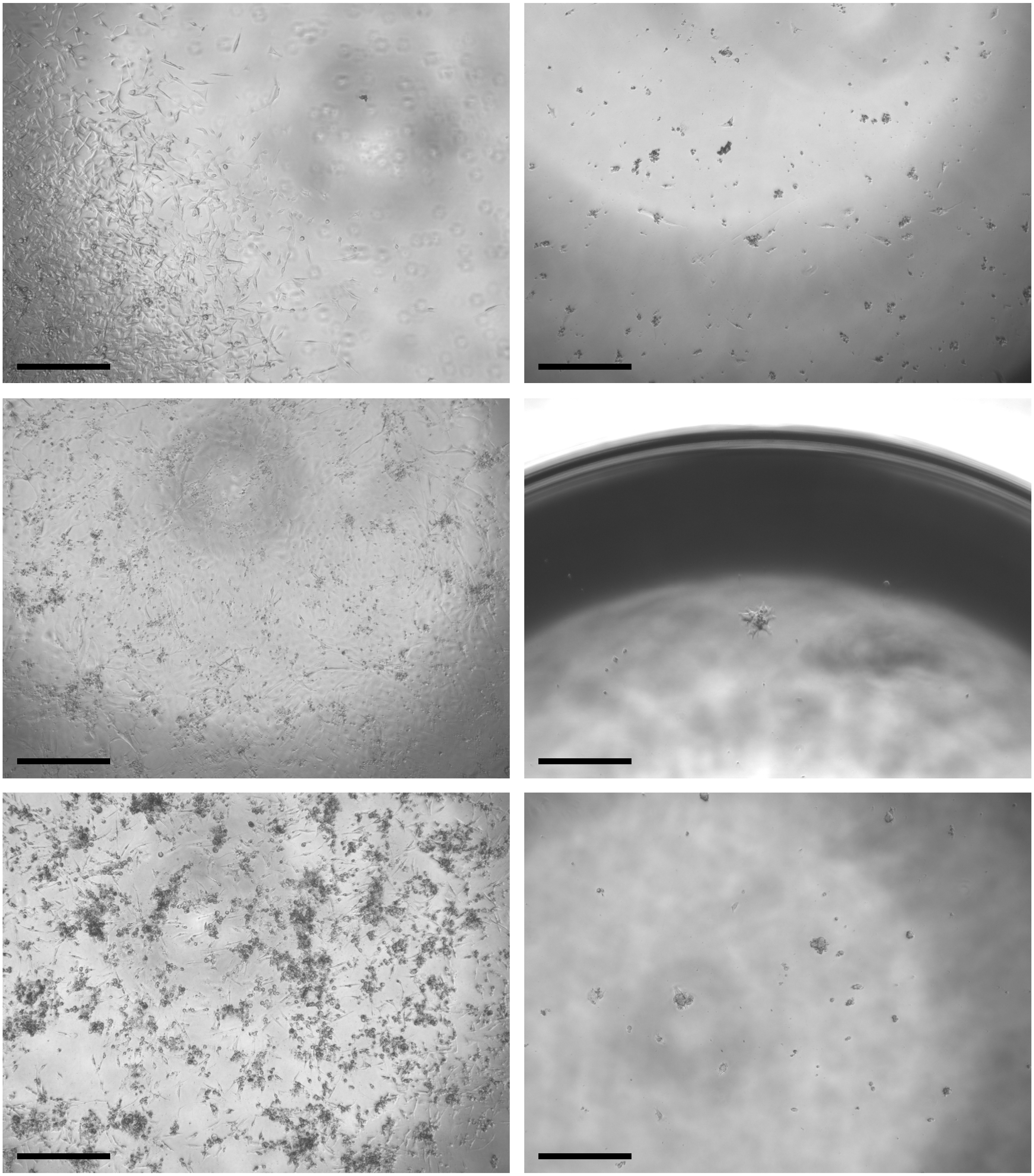

Supplementary Figure 11. Clonogenic Assays. Examples of positive (left) and negative (right) wells seeded with 786-0 cells are shown. Scale bars = 100 µm.
